# Decarboxylative trifluoromethylthiolation of pyridylacetates

**DOI:** 10.3762/bjoc.17.23

**Published:** 2021-01-25

**Authors:** Ryouta Kawanishi, Kosuke Nakada, Kazutaka Shibatomi

**Affiliations:** 1 Department of Applied Chemistry and Life Science, Toyohashi University of Technology, 1-1 Hibarigaoka, Tempaku-cho, Toyohashi 441-8580, Japan

**Keywords:** decarboxylation, fluorinated compounds, pyridine compounds, trifluoromethylthiolation

## Abstract

Decarboxylative trifluoromethylthiolation of lithium pyridylacetates was achieved using *N*-(trifluoromethylthio)benzenesulfonimide as the electrophilic trifluoromethylthiolation reagent. The reaction afforded the corresponding trifluoromethyl thioethers in good yield. Furthermore, the preparation of lithium pyridylacetates by saponification of the corresponding methyl esters and subsequent decarboxylative trifluoromethylthiolation were performed in a one-pot fashion.

## Introduction

The pyridine ring is found in numerous biologically active compounds. Therefore, efficient methods for synthesizing substituted pyridines are in high demand in pharmaceutical and agricultural chemistry [1–2]. Because of the unique features of fluorine atoms, fluorinated functional groups have also been recognized as important substructures in the design of medicinally relevant compounds [3–6]. Introducing a trifluoromethylthio group (CF_3_S–), which has high lipophilicity and strong electron-withdrawing properties, into medicinal compounds can improve their pharmacokinetic properties [7–11]. Hence, the development of a synthetic method for the preparation of trifluoromethyl thioethers has recently attracted much attention [12–15].

**Scheme 1:**
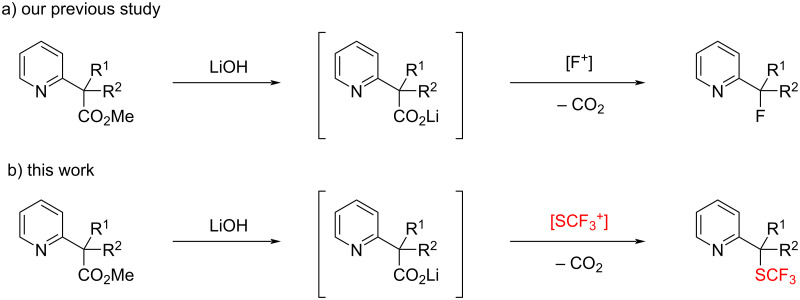
Electrophilic decarboxylative functionalization of 2-pyridylacetates.

Previously, our research group achieved decarboxylative functionalization of tertiary β-ketocarboxylic acids by exploiting their special ability to readily undergo decarboxylation [16–21]. During the course of this study, we found that lithium pyridylacetates undergo decarboxylative fluorination upon treatment with an electrophilic fluorination reagent to afford fluoromethylpyridines under catalyst-free conditions. Furthermore, we demonstrated the one-pot synthesis of fluoromethylpyridines from methyl pyridylacetates by saponification of methyl esters and subsequent decarboxylative fluorination (Scheme 1a) [21]. Herein, we describe the application of this method to decarboxylative trifluoromethylthiolation with an electrophilic trifluoromethylthiolation reagent (Scheme 1b) [22], which enables easy installation of the trifluoromethylthio group at a pyridylic carbon.

## Results and Discussion

First, we synthesized lithium 2-pyridylacetate **1a** according to our previously reported procedure [21] and subjected it to decarboxylative trifluoromethylthiolation with *N*-trifluoromethylthiosuccinimide (**4**) in DMF at room temperature for 15 h. However, the desired product **2a** was not observed (Table 1, entry 1). The use of *N*-trifluoromethylthiophthalimide (**5**) did not afford **2a** either (Table 1, entry 2). Fortunately, the use of *N*-(trifluoromethylthio)dibenzenesulfonimide **6** [23] gave **2a** in 14% yield, along with the protonated product **3a** in 31% yield (Table 1, entry 3). The yield of **2a** could be improved to 30% by adding MS 4 Å to the reaction mixture (Table 1, entry 4). Screening of various solvents revealed that THF was the best choice for this reaction (Table 1, entries 4–11), and the yield of **2a** was dramatically improved to 89% (Table 1, entry 11). In the absence of MS 4 Å, the yield of **2a** was diminished even when the reaction was carried out in THF (Table 1, entry 12).

**Table 1:** Screening of reaction conditions.

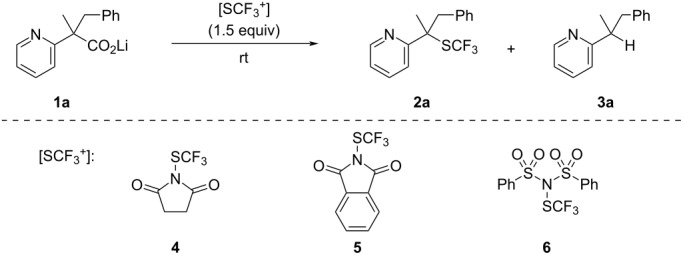					

entry	[SCF_3_^+^]	solvent	time (h)	yield of **2a** (%)	yield of **3a** (%)

1	**4**	DMF	15	0	0
2	**5**	DMF	72	0	0
3	**6**	DMF	3	14	31
4^a^	**6**	DMF	5	30	34
5^a^	**6**	DMSO	5	64	21
6^a^	**6**	acetonitrile	8	77	0
7^a^	**6**	toluene	168	72	0
8^a^	**6**	CH_2_Cl_2_	72	54	0
9^a^	**6**	*t*-BuOMe	72	55	0
10^a^	**6**	1,4-dioxane	9	75	0
11^a^	**6**	THF	8	89	0
12	**6**	THF	8	63	26
13^a,b^	**6**	THF	8	70	0

^a^The reaction was carried out with MS 4 Å (180 mg/0.2 mmol); ^b^1.1 equiv of **6** was used.

**Scheme 2:**
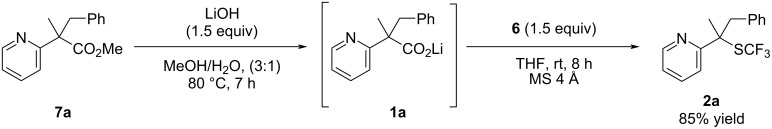
One-pot procedure for the synthesis of **2a**.

With the optimized reaction conditions in hand, we examined the one-pot synthesis of **2a** from methyl ester **7a**. Methyl 2-pyridylacetate **7a** were saponified with lithium hydroxide in a MeOH/H_2_O system. After completion of the reaction, the solvents were evaporated under reduced pressure. Then, THF, MS 4 Å, and **6** were added to the residue, and the mixture was stirred at room temperature for 8 h. This reaction successfully afforded the desired product **2a** in 85% yield over two steps (Scheme 2).

**Scheme 3:**
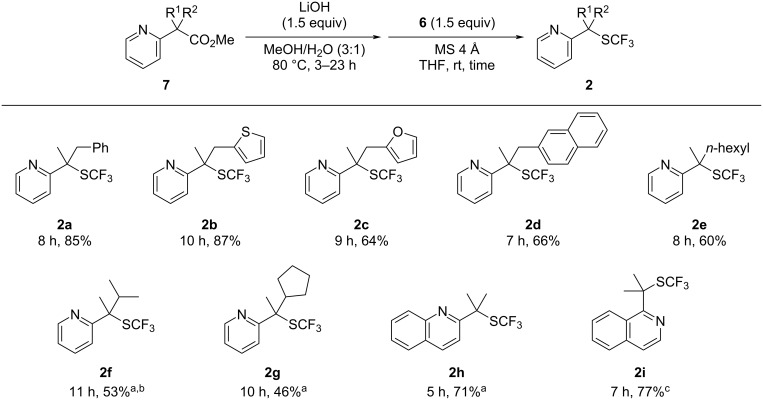
Substrate scope. ^a^Saponification was carried out with 2.5 equiv of LiOH, and 2.5 equiv of **6** was used for trifluoromethylthiolation. ^b^Saponification of **7** was carried out for 39 h. ^c^Saponification was carried out with 2.5 equiv of LiOH under reflux conditions, and 2.5 equiv of **6** was used for trifluoromethylthiolation.

**Scheme 4:**
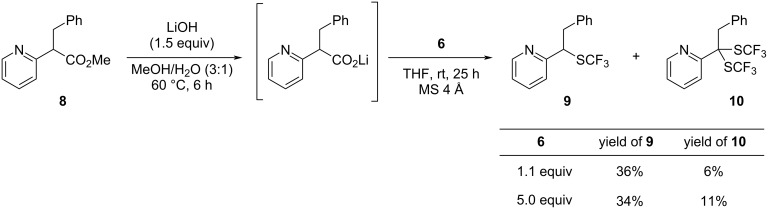
Reaction of α-monosubstituted 2-pyridylacetates.

Encouraged by the aforementioned result, we applied this method to several 2-pyridylacetates (Scheme 3). Methyl 2-pyridylacetates **7b–d** with arylmethyl substituents furnished the corresponding trifluoromethylthiolated products **2b−d** in good yields. α,α-Dialkyl-2-pyridylacetates **7e–g** also gave the desired products **2e–g** in moderate yields. The method could also be applied to substrates with quinoline and isoquinoline backbones to afford the corresponding products **2h** and **2i**. In addition, the reaction of α-monosubstituted 2-pyridylacetate **8** was performed to yield the corresponding mono-trifluoromethylthiolated product **9** in 36% yield, along with 6% yield of disubstituted product **10** (Scheme 4). Increasing the amount of **6** did not improve the yield of products **9** and **10** significantly.

**Scheme 5:**
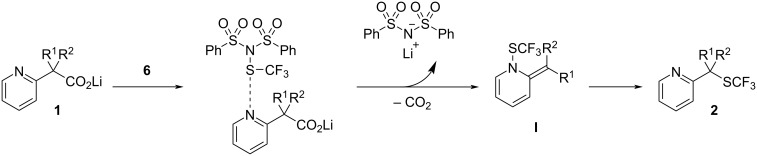
Proposed reaction pathway.

**Scheme 6:**
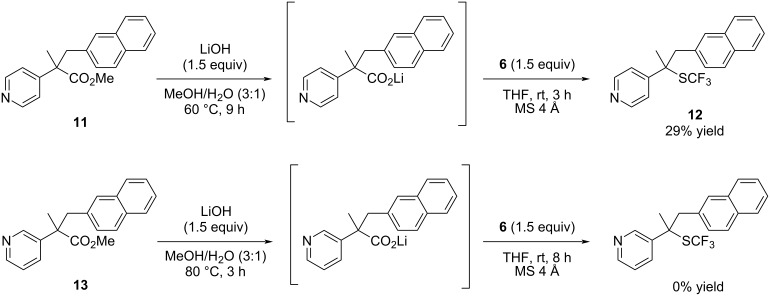
Reaction of 3- and 4-pyridylacetates.

Based on the abovementioned results and our previous study on decarboxylative fluorination [21], we propose a plausible mechanism for this reaction, as outlined in Scheme 5. An electrophilic sulfur atom of **6** approaches the nitrogen atom on the pyridine ring to promote decarboxylation via the formation of *N*-trifluoromethylthio-2-alkylidene-1,2-dihydropyridine intermediate **I**, which immediately isomerizes to afford **2** (Scheme 5). Methyl 4-pyridylacetate **11** also gave the corresponding trifluoromethylthiolated product **12** in 29% yield (Scheme 6), where the reaction was assumed to proceed via the *N*-trifluoromethylthio-4-alkylidene-1,4-dihydropyridine intermediate. In contrast, methyl 3-pyridylacetate **13** did not yield the trifluoromethylthiolated product at all, despite complete saponification of the methyl ester.

## Conclusion

In conclusion, we demonstrated the decarboxylative trifluoromethylthiolation of lithium 2- and 4-pyridylacetates to synthesize pyridine derivatives with a trifluoromethylthio group at a tertiary carbon center adjacent to the pyridine ring. Furthermore, saponification of methyl pyridylacetates and subsequent decarboxylative trifluoromethylthiolation of the resulting lithium salts were performed in a one-pot fashion. This method can easily convert an ester group into a trifluoromethylthio group. The resulting trifluoromethyl thioethers would be useful for the preparation of various medicinally relevant compounds.

## Supporting Information

File 1 Experimental procedures, characterization data, and copies of NMR spectra.
