# Supplementary material for: Decarboxylative trifluoromethylthiolation of pyridylacetates
Source: Beilstein J Org Chem. 2021 Jan 25;17:229–33. doi: 10.3762/bjoc.17.23 (PMC7849234; doi:10.3762/bjoc.17.23)
Supplement: File 1 — Experimental procedures, characterization data, and copies of NMR spectra. [file Beilstein_J_Org_Chem-17-229-s001.pdf]

BEILSTEIN JOURNAL OF ORGANIC CHEMISTRY

# Supporting Information

for

# Decarboxylative trifluoromethylthiolation of pyridylacetates

Ryouta Kawanishi, Kosuke Nakada and Kazutaka Shibatomi

*Beilstein J. Org. Chem.* **2021,** *17,* 229–233. doi:10.3762/bjoc.17.23

# Experimental procedures, characterization data, and copies of NMR spectra

License and Terms: This is a supporting information file under the terms of the Creative Commons Attribution License (https://creativecommons.org/licenses/by/4.0). Please note that the reuse, redistribution and reproduction in particular requires that the author(s) and source are credited and that individual graphics may be subject to special legal provisions.
The license is subject to the *Beilstein Journal of Organic Chemistry* terms and conditions: (https://www.beilstein-journals.org/bjoc/terms)

**Table of Contents**


1. **General information**......S1
2. **Material information**......S1
3. **Synthesis of methyl 2-pyridylacetate 7c**......S1
4. **Decarboxylative trifluoromethylthiolation of 2- and 4-pyridylacetates**......S2
5. **References** ......S7
6. **NMR spectra**......S8

## 1. General information

All non-aqueous reactions were carried out in dried glassware under an argon atmosphere and stirred using magnetic stir-plates. Thin-layer chromatography analyses were performed using pre-coated silica gel plates with a fluorescent indicator (F254) (Merck Millipore, Darmstadt, Germany). Visualization was accomplished by ultraviolet (UV) light (254 nm), phosphomolybdic acid, or *p*-anisaldehyde. Flash column chromatography was performed using silica gel 60 (mesh size 40–100) (Kanto Chemical Co., Inc. Tokyo, Japan). $^{1}$H, $^{13}$C, and $^{19}$F nuclear magnetic resonance (NMR) spectra were recorded on a JNM-ECX500 (500 MHz $^{1}$H, 126 MHz $^{13}$C, 470 MHz $^{19}$F) instrument (JEOL Ltd., Tokyo, Japan). Chemical shift values (δ) are reported in ppm (tetramethylsilane δ 0.00 for $^{1}$H; hexafluorobenzene δ −162.2 for $^{19}$F; residual chloroform δ 77.0 for $^{13}$C) unless otherwise noted. The high-resolution mass spectra (HRMS) were conducted on a JMS-T100TD time-of-flight mass spectrometer (DART) (JEOL Ltd.) or a micrOTOF-Q II HRMS/MS instrument with electrospray ionizer (ESI) (Bruker, Billerica, MA, USA).

## 2. Material information

Commercial grade reagents and solvents were used without further purification unless otherwise noted. Anhydrous dimethyl sulfoxide (DMSO), *tert*-butyl methyl ether, 1,4-dioxane, acetonitrile, MeOH, and dimethylformamide (DMF) were purchased from Sigma-Aldrich (St. Louis, MO, USA). Anhydrous toluene, dichloromethane and tetrahydrofurane (THF) were purchased from Kanto Chemical Co., Inc. and used after purification by a Glass Contour solvent dispensing system (Pure Process Technology, Nashua, NH, USA). 2-(bromomethyl)furan [1], *N*-(trifluoromethylthio)dibenzenesulfonimide **6** [2], *N*-(trifluoro-methylthio)succinimide (**4**), *N*-(trifluoromethylthio)phthalimide (**5**) [3], methyl 2-pyridylacetate **7**, **8**, methyl 4-pyridylacetate **11** and 3-pyridylacetate **13** [4] were prepared according to literature procedures.

## 3. Synthesis of methyl 2-pyridylacetate 7c

### Methyl 2-(pyridin-2-yl)-3-(furan-2-yl)propanoate (S1)

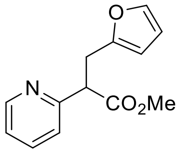


**S1**

Methyl 2-(pyridin-2-yl)acetate (1.5 g, 9.9 mmol) was added to a stirred suspension of NaH (60% in oil, washed by hexane) (429 mg, 10.7 mmol) in THF (17 mL) at 0 °C, and the mixture was stirred at 0 °C for 1 h. 2-(bromomethyl)furan (1.9 g, 11.9 mmol) in THF (3.0 mL) was added to the mixture, and the reaction mixture was stirred at room temperature for 13 h. After the reaction was completed, the reaction was quenched by adding $H_2O$ and extracted with ethyl acetate. The combined organic layer was dried over anhydrous $Na_2SO_4$ and concentrated. The crude product was purified by flash column chromatography (hexane/ethyl acetate = 2:1) on silica gel to give the title compound as a yellow oil (1.2 g, 54% yield).

**$^{1}$H NMR** (500MHz, $CDCl_3$): δ 8.59–8.57 (m, 1H), 7.62 (ddd, *J* = 7.6, 7.6, 1.9 Hz, 1H), 7.27 (dd, *J* = 1.9, 0.8 Hz, 1H), 7.22 (ddd, *J* = 7.6, 1.2, 1.2 Hz, 1H), 7.18 (ddd, *J* = 7.6, 5.0, 1.2 Hz, 1H), 6.21–6.20 (m, 1H), 5.92 (dd, *J* = 3.1, 0.8 Hz, 1H), 4.22 (t, *J* = 7.6 Hz, 1H), 3.69 (s ,3H), 3.50 (dd, *J* = 15.3, 7.6 Hz, 1H), 3.28 (dd, *J* = 15.3,

7.6 Hz, 1H). **$^{13}$C NMR** (126 MHz, $CDCl_3$): δ 172.2, 157.4, 152.4, 149.4, 141.1, 136.5, 122.7, 122.2, 110.0, 106.3, 52.3, 52.0, 30.2. **HRMS** (DART): $[M+H]^+$ calcd. for $C_{13}H_{14}N_1O_3$, 232.09715; found, 232.09737.

**Methyl 2-methyl-2-(pyridin-2-yl)-3-(furan-2-yl)propanoate (7c)**

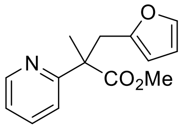


**7c**

Methyl 2-(pyridin-2-yl)-3-(furan-2-yl)propanoate (**S1**) (904 mg, 3.9 mmol) was added to a solution of LiHMDS (1.0 M in THF) (5.8 mL) in THF (7.9 mL) at 0 °C, and the mixture was stirred at 0 °C for 1 h. Then, iodomethane (0.36 mL, 5.8 mmol) was added to the mixture, and the mixture was stirred at room temperature for 14 h. After the reaction was completed, the reaction was quenched by adding saturated $NH_4Cl$ aqueous solution, and the mixture was extracted with ethyl acetate. The combined organic layer was dried over $Na_2SO_4$ and concentrated. The crude product was purified by flash column chromatography (hexane/ethyl acetate = 4:1) to provide the title compound as a yellow oil (674 mg, 71% yield).
**$^1$H NMR** (500 MHz, $CDCl_3$): δ 8.60–8.58 (m, 1H), 7.63 (ddd, $J$ = 7.6, 7.6, 1.9 Hz, 1H), 7.24–7.24 (m, 1H), 7.20–7.15 (m, 3H), 6.21 (dd, $J$ = 3.1, 1.9 Hz, 1H), 5.85-5.84 (m, 1H), 3.72 (s, 3H), 3.50 (d, $J$ = 14.5 Hz, 1H), 3.45 (d, $J$ = 14.5 Hz, 1H), 1.57 (s, 3H). **$^{13}$C NMR** (126 MHz, $CDCl_3$): δ 175.4, 162.0, 152.1, 148.9, 141.3, 136.4, 121.8, 120.4, 110.1, 107.8, 53.6, 52.2, 36.4, 22.3. **HRMS** (DART): $[M+H]^+$ calcd. for $C_{14}H_{16}N_1O_3$, 246.11302; found, 246.11275.

## 4. Decarboxylative trifluoromethylthiolation of 2-pyridylacetates

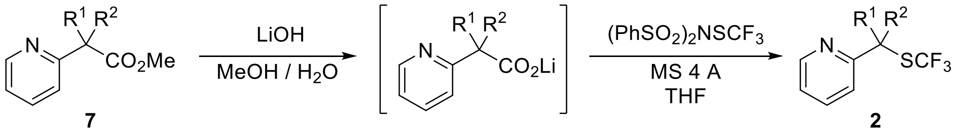


**7** **2**

**General procedure**: In a glass tube or flask containing magnetic stir bar was charged with lithium hydroxide and a solution of methyl 2-pyridylacetate **7** in MeOH and $H_2O$ (3:1, 0.2 M), then it was sealed with septum cap. After the reaction was completed, all solvents were removed, *N*-(trifluoromethylthio)dibenzene-sulfonimide, activated MS 4 Å (180 mg/0.2 mmol) and THF were added to the mixture, and the mixture was stirred until the reaction completed. The mixture was directly subjected to flash column chromatography to give the corresponding trifluoromethylthiolated product.

**2-(2-Trifluoromethylthio-1-phenylpropan-2-yl)pyridine (2a)**

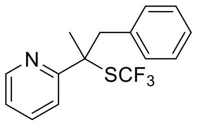


**2a**

The compound was prepared according to the **General procedure**. Saponification was performed with lithium hydroxide (7.3 mg, 0.30 mmol) and **7a** (51 mg, 0.20 mmol) in MeOH/$H_2O$ (0.72/0.24 mL) at 80 °C (bath temp.) for 7 h and solvents were removed at 120 °C. Decarboxylative trifluoromethylthiolation was performed with *N*-(trifluoromethylthio)dibenzenesulfonimide (123 mg, 0.31 mmol, 1.5 equiv) and activated MS 4 Å (182 mg) in THF (1.0 mL) at room temperature for 8 h. Flash column chromatography (hexane/diethyl ether = 9:1) gave the title compound as a colorless oil (50 mg, 85% yield).

$^1$**H NMR** (500 MHz, $CDCl_3$): δ 8.68–8.67 (m, 1H), 7.61 (ddd, *J* = 8.0, 8.0, 1.9 Hz, 1H), 7.31–7.30 (m, 1H), 7.21–7.11 (m, 4H), 6.76-6.79 (m, 2H), 3.66 (d, *J* = 13.2 Hz, 1H), 3.30 (d, *J* = 13.2 Hz, 1H), 1.92 (s, 3H). $^{13}$**C NMR** (126 MHz, $CDCl_3$): δ 161.1, 148.8, 136.3, 135.5, 130.6 (q, *J* = 308.3 Hz), 130.5, 127.8, 126.9, 122.3, 121.4, 58.3, 48.3, 25.0. $^{19}$**F NMR** (470 MHz, $CDCl_3$): δ –36.4. **HRMS** (DART): $[M+H]^+$ calcd. for $C_{15}H_{15}F_3N_1S_1$, 298.08773; found, 298.08796.

**2-(2-Trifluoromethylthio-1-(thiophen-2-yl)propan-2-yl)pyridine (2b)**

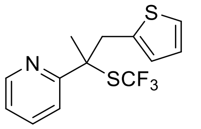

**2b**

The compound was prepared according to the **General procedure**. Saponification was performed with lithium hydroxide (7.3 mg, 0.30 mmol) and **7b** (53 mg, 0.20 mmol) in MeOH/$H_2O$ (0.72/0.24 mL) at 80 °C (bath temp.) for 23 h and solvents were removed at 110 °C. Decarboxylative trifluoromethylthiolation was performed with *N*-(trifluoromethylthio)dibenzenesulfonimide (122 mg, 0.31 mmol, 1.5 equiv) and activated MS 4 Å (180 mg) in THF (1.0 mL) at room temperature for 10 h. Flash column chromatography (hexane/diethyl ether = 9:1) gave the title compound as a colorless oil (53 mg, 87% yield).

$^1$**H NMR** (500 MHz, $CDCl_3$): 8.68 (ddd, *J* = 4.9, 1.9, 0.8 Hz, 1H), 7.66 (ddd, *J* = 7.8, 7.8, 1.9 Hz, 1H), 7.43–7.41 (m, 1H), 7.24–7.21 (m, 1H), 7.04 (dd, *J* = 5.3, 1.2 Hz, 1H), 6.82 (dd, *J* = 5.3, 3.4 Hz, 1H), 6.59 (d, *J* = 3.4 Hz, 1H), 4.00 (d, *J* = 14.1 Hz, 1H), 3.56 (d, *J* = 14.1 Hz, 1H), 1.94 (s, 3H). $^{13}$**C NMR** (126 MHz, $CDCl_3$): δ 160.6, 149.0, 137.4, 136.5, 130.5 (q, *J* = 308.3 Hz), 127.9, 126.3, 124.9, 122.6, 121.6, 57.8, 42.1, 25.1. $^{19}$**F NMR** (470 MHz, $CDCl_3$): δ –36.4. **HRMS** (DART): $[M+H]^+$ calcd. for $C_{13}H_{13}F_3N_1S_2$, 304.04415; found, 304.04440.

**2-(2-Trifluoromethylthio-1-(furan-2-yl)propan-2-yl)pyridine (2c)**

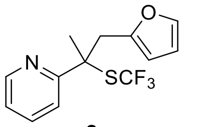

**2c**

The compound was prepared according to the **General procedure**. Saponification was performed with lithium hydroxide (6.5 mg, 0.27 mmol) and **7c** (45 mg, 0.18 mmol) in MeOH/$H_2O$ (0.65/0.22 mL) at 80 °C (bath temp.) for 3 h and solvents were removed at 120 °C. Decarboxylative trifluoromethylthiolation was performed with *N*-(trifluoromethylthio)dibenzenesulfonimide (110 mg, 0.28 mmol, 1.5 equiv) and activated MS 4 Å (166 mg) in THF (0.9 mL) at room temperature for 9 h. Flash column chromatography (hexane/diethyl ether = 9:1) gave the title compound as a yellow oil (34 mg, 64% yield).

$^1$**H NMR** (500 MHz, $CDCl_3$): δ 8.64–8.63 (m, 1H), 7.66 (ddd, *J* = 7.8, 7.8, 1.9 Hz, 1H), 7.44–7.42 (m, 1H), 7.23–7.19 (m, 2H), 6.19 (dd, *J* = 3.1, 1.9 Hz, 1H), 5.79 (d, *J* = 3.1 Hz, 1H), 3.69 (d, *J* = 14.9 Hz, 1H), 3.45 (d, *J* = 14.9 Hz, 1H), 1.95 (s, 3H). $^{13}$**C NMR** (126 MHz, $CDCl_3$): δ 161.1, 150.5, 148.8, 141.7, 136.4, 130.5 (q, *J* = 308.3 Hz), 122.4, 121.1, 110.2, 108.9, 57.5, 40.5, 25.5. $^{19}$**F NMR** (470 MHz, $CDCl_3$): δ –36.6. **HRMS** (DART): $[M+H]^+$ calcd. for $C_{13}H_{13}F_3N_1O_1S_1$, 288.06699; found, 288.06693

**2-(2-Trifluoromethylthio-1-(naphthalen-2-yl)propan-2-yl)pyridine (2d)**

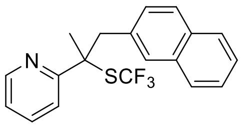

**2d**

The compound was prepared according to the **General procedure**. Saponification was performed with lithium hydroxide (7.3 mg, 0.30 mmol) and **7d** (62 mg, 0.20 mmol) in MeOH/$H_2O$ (0.72/0.24 mL) at 80 °C (bath temp.) for 8 h and solvents were evaporated under vacuum at 80 °C. Decarboxylative trifluoromethylthiolation was performed with *N*-(trifluoromethylthio)dibenzenesulfonimide (121 mg, 0.30 mmol, 1.5 equiv) and activated MS 4 Å (181 mg) in THF (1.0 mL) at room temperature for 7 h. Flash column chromatography (hexane/diethyl ether = 20:1 to 10:1) gave the title compound as a colorless oil (46 mg, 66% yield).
**$^1$H NMR** (500 MHz, $CDCl_3$): δ 8.72–8.71 (m, 1H), 7.75–7.63 (m, 2H), 7.60–7.56 (m, 2H), 7.42–7.40 (m, 2H), 7.28–7.20 (m, 3H), 6.82–6.80 (m, 1H), 3.84 (d, *J* = 13.4 Hz, 1H), 3.46 (d, *J* = 13.4 Hz, 1H), 1.96 (s, 3H). **$^{13}$C NMR** (126 MHz, $CDCl_3$): δ 161.1, 148.9, 136.3, 133.2, 133.0, 132.3, 130.6 (q, *J* = 308.3 Hz), 129.5, 128.5, 127.6, 127.5, 127.3, 125.9, 125.7, 122,4, 121.5, 58.5, 48.5, 25.1. **$^{19}$F NMR** (470 MHz, $CDCl_3$): δ –36.3. **HRMS** (DART): $[M+H]^+$ calcd. for $C_{19}H_{17}F_3N_1S_1$, 348.10332; found, 348.10338.

**2-(2-Trifluoromethylthiooctan-2-yl)pyridine (2e)**

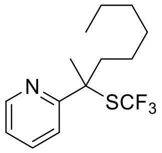

**2e**

The compound was prepared according to the **General procedure**. Saponification was performed with lithium hydroxide (7.4 mg, 0.31 mmol) and **7e** (51 mg, 0.20 mmol) in MeOH/$H_2O$ (0.72/0.24 mL) at 80 °C (bath temp.) for 12 h and solvents were removed at 120 °C. Decarboxylative trifluoromethylthiolation was performed with *N*-(trifluoromethylthio)dibenzenesulfonimide (122 mg, 0.31 mmol, 1.5 equiv) and activated MS 4 Å (180 mg) in THF (1.0 mL) at room temperature for 9 h. Flash column chromatography (hexane/diethyl ether = 9:1) gave the title compound as a colorless oil (36 mg, 60% yield).
**$^1$H NMR** (500 MHz, $CDCl_3$): δ 8.60–8.58 (m, 1H), 7.68 (ddd, *J* = 7.7, 7.7, 1.9 Hz, 1H), 7.54–7.52 (m, 1H), 7.19–7.16 (m, 1H), 2.23–2.18 (m, 1H), 2.05–1.92 (m, 1H), 1.95 (s, 3H), 1.30–1.16 (m, 7H), 1.11–1.05 (m, 1H), 0.85–0.83 (m, 3H). **$^{13}$C NMR** (126 MHz, $CDCl_3$): δ 162.2, 148.7, 136.4, 130.6 (q, *J* = 308.3 Hz), 122.0, 120.9, 58.4, 42.0, 31.5, 29.3, 26.0, 24.3, 22.5, 14.0. **$^{19}$F NMR** (470 MHz, $CDCl_3$): δ –36.7. **HRMS** (DART): $[M+H]^+$ calcd. for $C_{14}H_{21}F_3N_1S_1$, 292.13468; found, 292.13496.

**2-(3-Methyl-2-((trifluoromethyl)thio)butan-2-yl)pyridine (2f)**

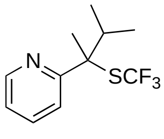

**2f**

In a 20 mL flask containing magnetic stir bar and equipped with reflux condenser was charged with lithium hydroxide (10.4 mg, 0.44 mmol) and a solution of methyl 2-pyridylacetate **7f** (36 mg, 0.17 mmol) in MeOH and $H_2O$ (0.63/0.21 mL). After the reaction mixture was stirred for 39 h under reflux condition, all solvents were removed under vacuum at 60 °C. Decarboxylative trifluoromethylthiolation was performed with *N*-(trifluoromethylthio)dibenzenesulfonimide (174 mg, 0.44 mmol, 2.5 equiv) and activated MS 4 Å (203 mg) in

THF(1.8 mL) at room temperature for 11 h. Flash column chromatography (hexane/diethyl ether = 4:1) gave the title compound as a colorless oil (23 mg, 53% yield).
**$^1$H NMR** (500 MHz, $CDCl_3$): δ 8.61–8.59 (m, 1H), 7.67 (ddd, *J* = 8.0, 8.0, 1.9 Hz, 1H), 7.59 (d, *J* = 8.0 Hz, 1H), 7.18–7.15 (m, 1H), 2.56 (sep, *J* = 6.9 Hz, 1H), 1.91 (s, 3H), 1.10 (d, *J* = 6.9 Hz, 3H), 0.68 (d, *J* = 6.9 Hz, 3H). **$^{13}$C NMR** (126 MHz, $CDCl_3$): δ 162.6, 148.5, 136.1, 130.7 (q, *J* = 308.3 Hz), 121.8, 121.7, 62.2, 37.8, 19.9, 18.0, 17.5. **$^{19}$F NMR** (470 MHz, $CDCl_3$): δ –36.3. **HRMS** (ESI): $[M+H]^+$ calcd. for $C_{11}H_{15}F_3N_1S_1$, 250.0872; found, 250.0875.

**2-(1-Cyclopentyl-1-((trifluoromethyl)thio)ethyl)pyridine (2g)**

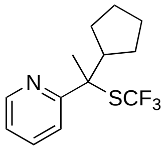


**2g**

The compound was prepared according to the **General procedure**. Saponification was performed with lithium hydroxide (12.5 mg, 0.52 mmol) and **7g** (49 mg, 0.21 mmol) in MeOH/$H_2O$ (0.75/0.25 mL) under reflux condition for 16.5 h and solvents were evaporated under vacuum at 60 °C. Decarboxylative trifluoromethylthiolation was performed with *N*-(trifluoromethylthio)dibenzenesulfonimide (207 mg, 0.52 mmol, 2.5 equiv) and activated MS 4 Å (189 mg) in THF (2.0 mL) at room temperature for 10 h. Flash column chromatography (hexane/diethyl ether = 4:1) gave the title compound as a colorless oil (28 mg, 46% yield).
**$^1$H NMR** (500 MHz, $CDCl_3$): δ 8.60–8.58 (m, 1H), 7.68–7.63 (m, 2H), 7.17–7.14 (m, 1H), 2.70 (quin, *J* = 8.6 Hz, 1H), 1.96 (s, 3H), 1.80–1.73 (m, 1H), 1.63–1.23 (m, 7H). **$^{13}$C NMR** (126 MHz, $CDCl_3$): δ 162.7, 148.4, 136.1, 130.6 (q, *J* = 308.3 Hz), 121.8, 121.6, 60.9, 50.0, 27.9, 27.5, 25.8, 25.4, 21.6. **$^{19}$F NMR** (470 MHz, $CDCl_3$): δ –36.3. **HRMS** (ESI): $[M+H]^+$ calcd. for $C_{13}H_{17}F_3N_1S_1$, 276.1028; found, 276.1039.

**2-(2-Trifluoromethylthiopropan-2-yl)quinoline (2h)**

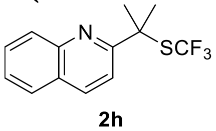


**2h**

The compound was prepared according to the **General procedure**. Saponification was performed with lithium hydroxide (10.4 mg, 0.44 mmol) and **7h** (40 mg, 0.17 mmol) in MeOH/$H_2O$ (0.63/0.20 mL) at 80 °C (bath temp.) for 4 h and solvents were evaporated under vacuum at 60 °C. Decarboxylative trifluoromethylthiolation was performed with *N*-(trifluoromethylthio)dibenzenesulfonimide (173 mg, 0.44 mmol, 2.5 equiv) and activated MS 4 Å (180 mg) in THF (1.8 mL) at room temperature for 5 h. Flash column chromatography (hexane/diethyl ether = 10:1) gave the title compound as a colorless oil (34 mg, 71% yield).
**$^1$H NMR** (500 MHz, $CDCl_3$): δ 8.15 (d, *J* = 8.8 Hz, 1H), 8.08 (d, *J* = 8.4 Hz, 1H), 7.86 (d, *J* = 8.4 Hz, 1H), 7.80 (d, *J* = 8.0 Hz, 1H), 7.77 (d, *J* = 8.8 Hz, 1H), 7.71 (ddd, *J* = 8.4, 7.1, 1.5 Hz, 1H), 2.00 (s, 6H). **$^{13}$C NMR** (126 MHz, $CDCl_3$): δ 162.8, 146.9, 136.5, 130.4 (q , *J* = 308.3 Hz), 129.6, 129.5, 127.3, 126.8, 126.7, 118.7, 55.1, 29.7.**$^{19}$F NMR** (470 MHz, $CDCl_3$): δ –37.2. **HRMS** (ESI): $[M+H]^+$ calcd. for $C_{13}H_{13}F_3N_1S_1$, 272.0715; found,272.0706.

**1-(2-Trifluoromethylthiopropan-2-yl)isoquinoline (2i)**

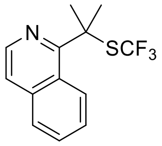


**2i**

The compound was prepared according to the **General procedure**. Saponification was performed with lithium hydroxide (10.5 mg, 0.46 mmol) and **7i** (40 mg, 0.17 mmol) in MeOH/$H_2O$ (0.63/0.20 mL) under reflux for 16 h and solvents were evaporated under vacuum at 60 °C. Decarboxylative trifluoromethylthiolation was performed with *N*-(trifluoromethylthio)dibenzenesulfon-imide (173 mg, 0.44 mmol, 2.5 equiv) and activated MS 4 Å (180 mg) in THF (1.8 mL) at room temperature for 7 h. Flash column chromatography (hexane/diethyl ether = 9:1) gave the title compound as a yellow oil (36 mg, 77% yield).

**$^1$H NMR** (500 MHz, $CDCl_3$): δ 8.81 (d, *J* = 8.8 Hz, 1H), 8.45 (d, *J* = 5.4 Hz, 1H), 7.86 (d, *J* = 8.0 Hz, 1H), 7.69–7.59 (m, 3H), 2.14 (s, 6H). **$^{13}$C NMR** (126 MHz, $CDCl_3$): δ 160.0, 140.2, 137.5, 130.4 (q, *J* = 308.3 Hz), 129.3, 128.2, 127.1, 126.2, 125.6, 121.5, 55.6, 31.1. **$^{19}$F NMR** (470 MHz, $CDCl_3$): δ –37.1. **HRMS** (DART): $[M+H]^+$ calcd. for $C_{13}H_{13}F_3N_1S_1$, 272.07208; found, 272.07234.

**2-(2-Phenyl-1-((trifluoromethyl)thio)ethyl)pyridine (9)**

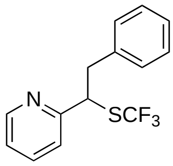


**9**

The compound was prepared according to the **General procedure**. Saponification was performed with lithium hydroxide (6.9 mg, 0.29 mmol) and **8** (46 mg, 0.19 mmol) in MeOH/$H_2O$ (0.69/0.23 mL) at 60 °C (bath temp.) and solvents were evaporated under vacuum at 60 °C. Decarboxylative trifluoromethylthiolation was performed with *N*-(trifluoromethylthio)dibenzenesulfonimide (83 mg, 0.21 mmol, 1.1 equiv) and activated MS 4 Å (172 mg) in THF (1.9 mL) at room temperature for 25 h. Flash column chromatography (hexane/diethyl ether = 8:1 to 2:1) gave the title compound as a colorless oil (19 mg, 36% yield) along with ditrifluoromethylthiolated compound (6% yield).

**$^1$H NMR** (500 MHz, $CDCl_3$): δ 8.63–8.61 (m, 1H), 7.53 (ddd, *J* = 7.6, 7.6, 1.9 Hz, 1H), 7.21–7.16 (m, 4H), 6.98–6.95 (m, 3H), 4.57 (dd, *J* = 9.2, 6.1 Hz, 1H), 3.46–3.36 (m, 2H). **$^{13}$C NMR** (126 MHz, $CDCl_3$): δ158.6, 149.8, 137.3, 136.4, 130.8 (q, *J* = 307.1 Hz), 129.2, 128.4, 126.8, 1228, 122.7, 51.9, 42.3. **$^{19}$F NMR** (470 MHz, $CDCl_3$): δ –40.6. **HRMS** (ESI): $[M+H]^+$ calcd. for $C_{14}H_{13}F_3N_1S_1$, 284.0715; found, 284.0710.

**4-(2-trifluoromethylthio-1-(naphthalen-2-yl)propan-2-yl)pyridine (12)**

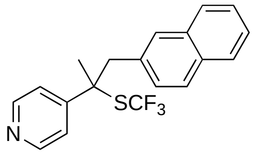


**12**

The compound was prepared according to the **General procedure**. Saponification was performed with lithium hydroxide (7.1 mg, 0.30 mmol) and **11** (60 mg, 0.20 mmol) in MeOH/$H_2O$ (0.72/0.24 mL) at 60°C (bath temp.) for 9 h and solvents were evaporated under vacuum at 60 °C. Decarboxylative trifluoromethylthiolation was performed with *N*-(trifluoromethylthio)dibenzenesulfonimide (118 mg, 0.30 mmol, 1.5 equiv) and activated

MS 4 Å (181 mg) in THF (1.0 mL) at room temperature for 3 h. Flash column chromatography (diethyl ether) gave the title compound as a yellow oil (20 mg, 29% yield).
$^{1}$**H NMR** (500 MHz, $CDCl_3$): δ 8.52 (d, *J* = 5.7 Hz, 2H), 7.78–7.75 (m, 1H), 7.69–7.66 (m, 1H), 7.62 (d, *J* = 8.4 Hz, 1H), 7.46–7.42 (m, 2H), 7.38–7.37 (m, 2H), 7.29 (br, 1H), 6.82 (dd, *J* = 8.4, 1.9 Hz, 1H), 3.42 (d, *J* = 13.4 Hz, 1H), 3.32 (d, *J* = 13.4 Hz, 1H), 1.94 (s, 3H). $^{13}$**C NMR** (126 MHz, $CDCl_3$): δ 151.2, 149.8, 132.9, 132.4, 131.5, 130.0 (q, *J* = 308.3 Hz), 129.7, 128.4, 127.7, 127.6, 127.5, 126.2, 126.1, 122.3, 55.8, 49.2, 25.6 $^{19}$**F NMR** (470 MHz, $CDCl_3$): δ –36.1. **HRMS** (ESI): $[M+H]^+$ calcd. for $C_{19}H_{17}F_3N_1S_1$, 348.1028; found, 348.1035.

## 5. References


1. Guo, Y.; Quan, T.; Lu, Y.; Luo, T. *J. Am. Chem. Soc.* **2004**, *139*, 6815–6818.
2. Liu, X.; An, R.; Zhang, X.; Luo, J.; Zhao, X. *Angew. Chem. Int. Ed.* **2016**, *55*, 5846–5850.
3. Kang, K.; Xu, C.; Shen, Q. *Org. Chem. Front.* **2014**, *1*, 294–297.
4. Kawanishi, R.; Phongphane, L.; Iwasa, S.; Shibatomi, K. *Chem. Eur. J.* **2019**, *25*, 7453–7456.

## 6. NMR spectra

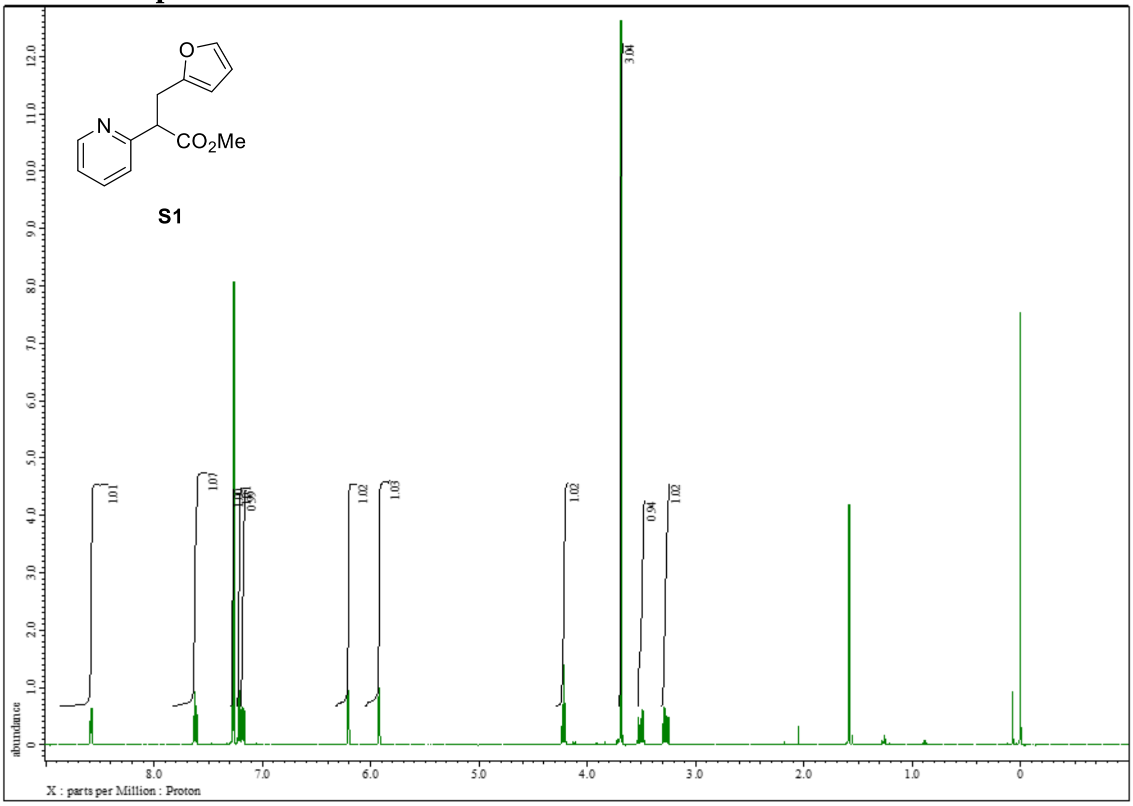


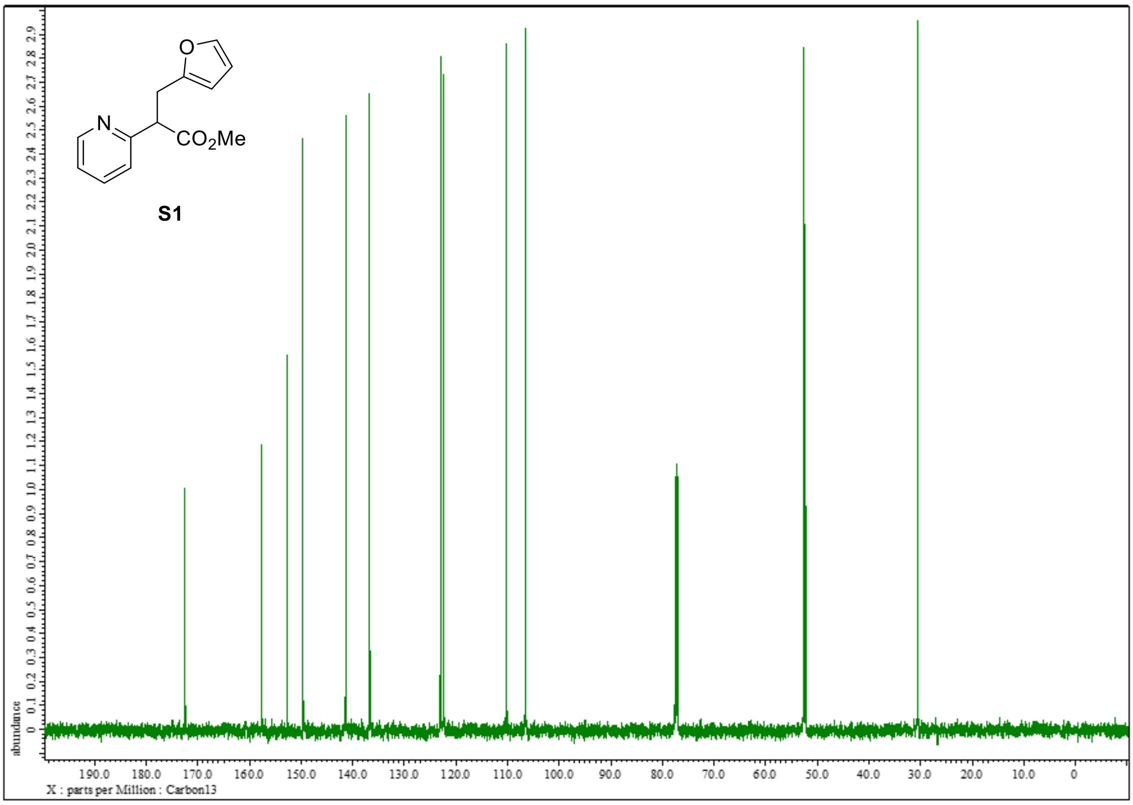

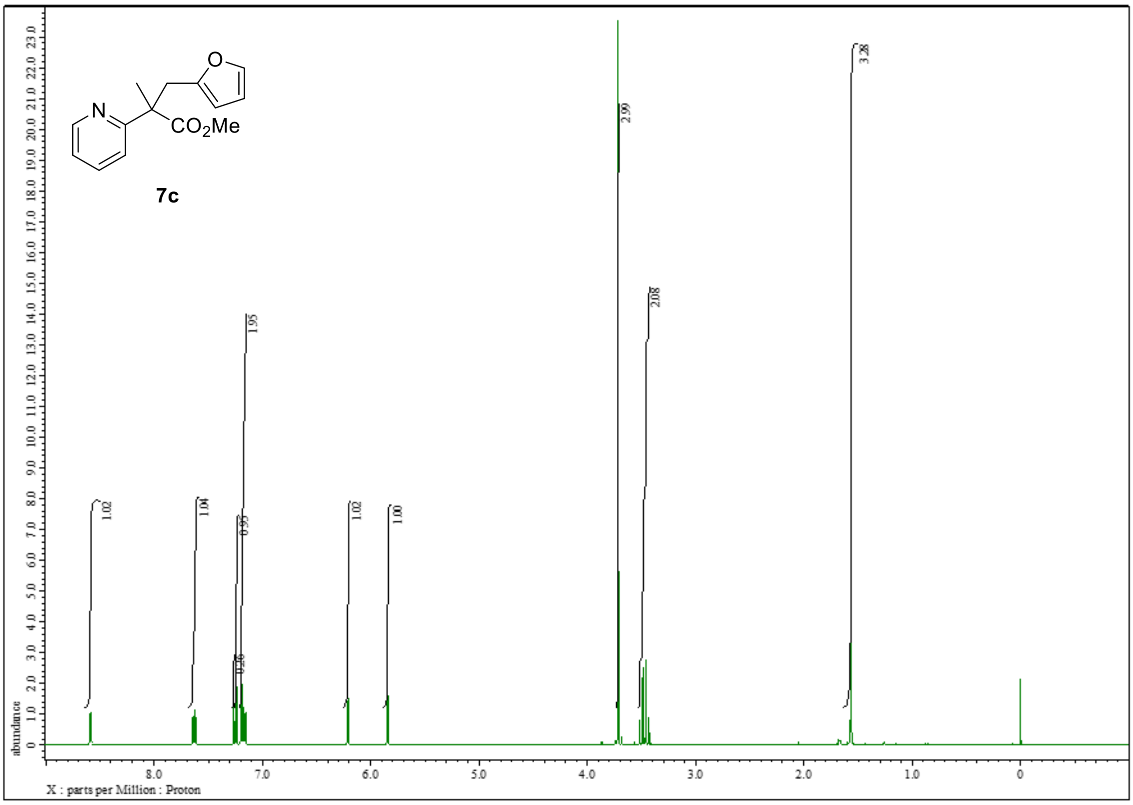

7c
abundance
X : parts per Million : Proton


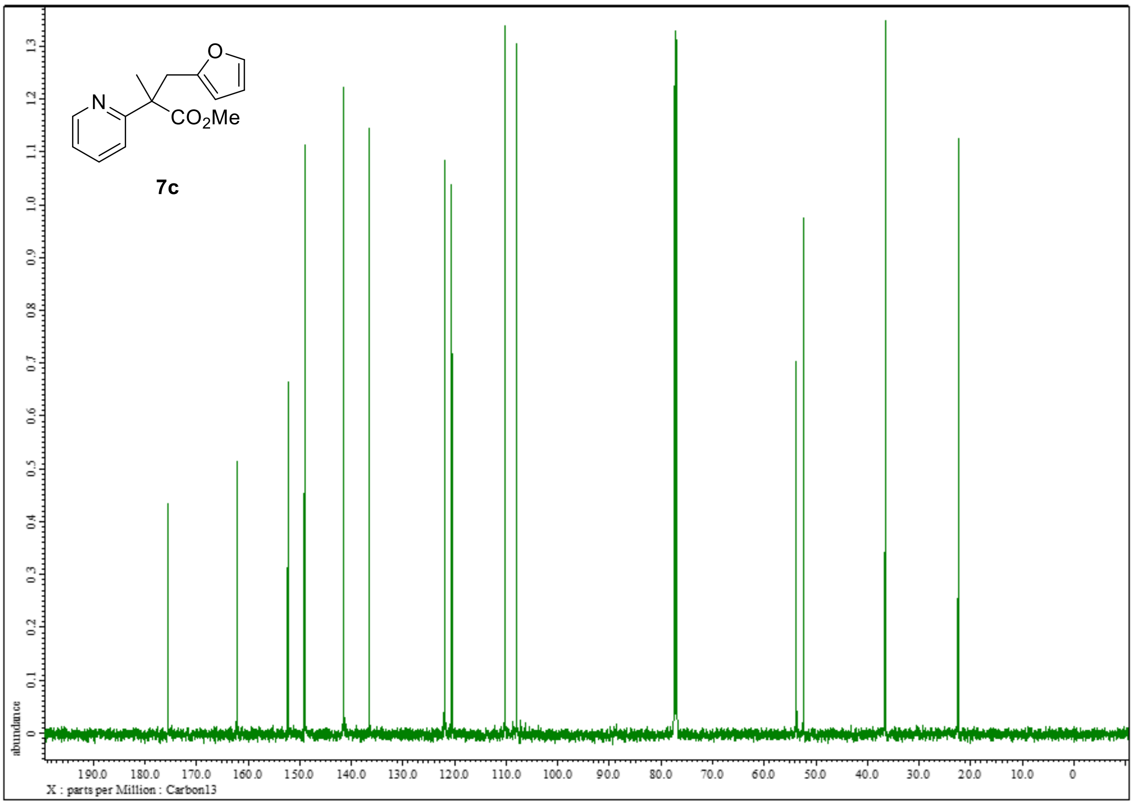

7c
abundance
X : parts per Million : Carbon13

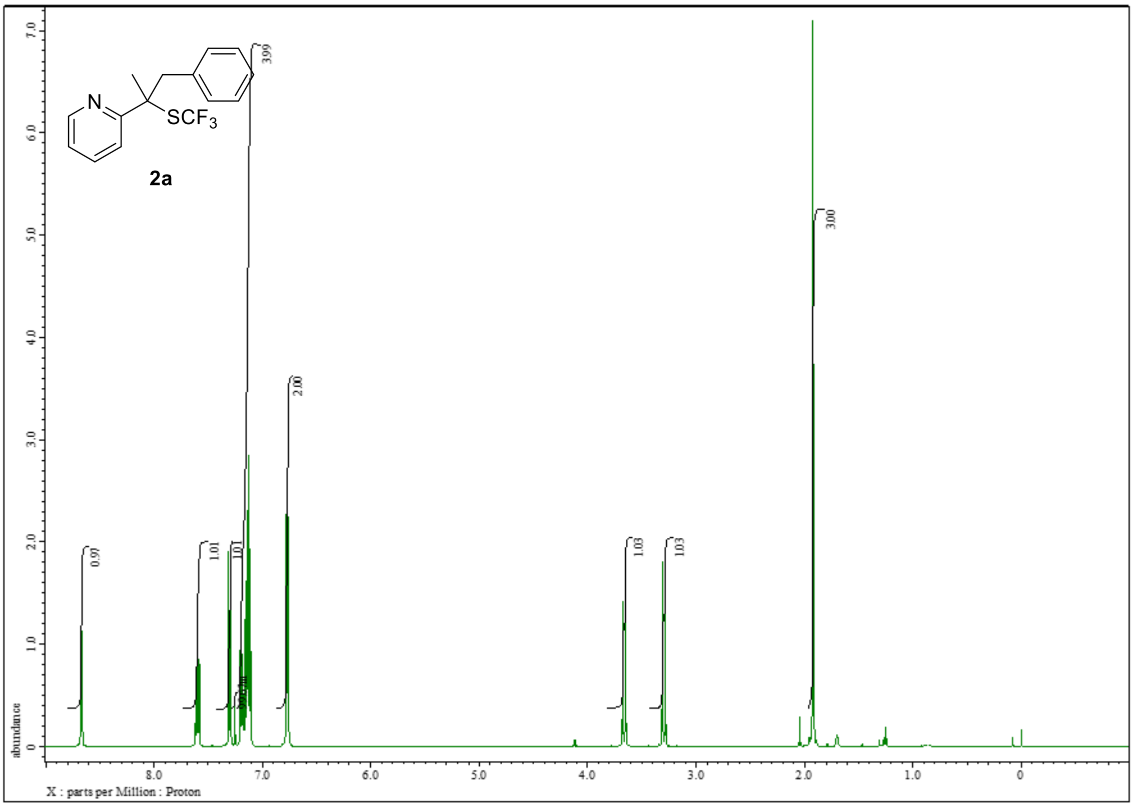
2a
X : parts per Million : Proton
abundance

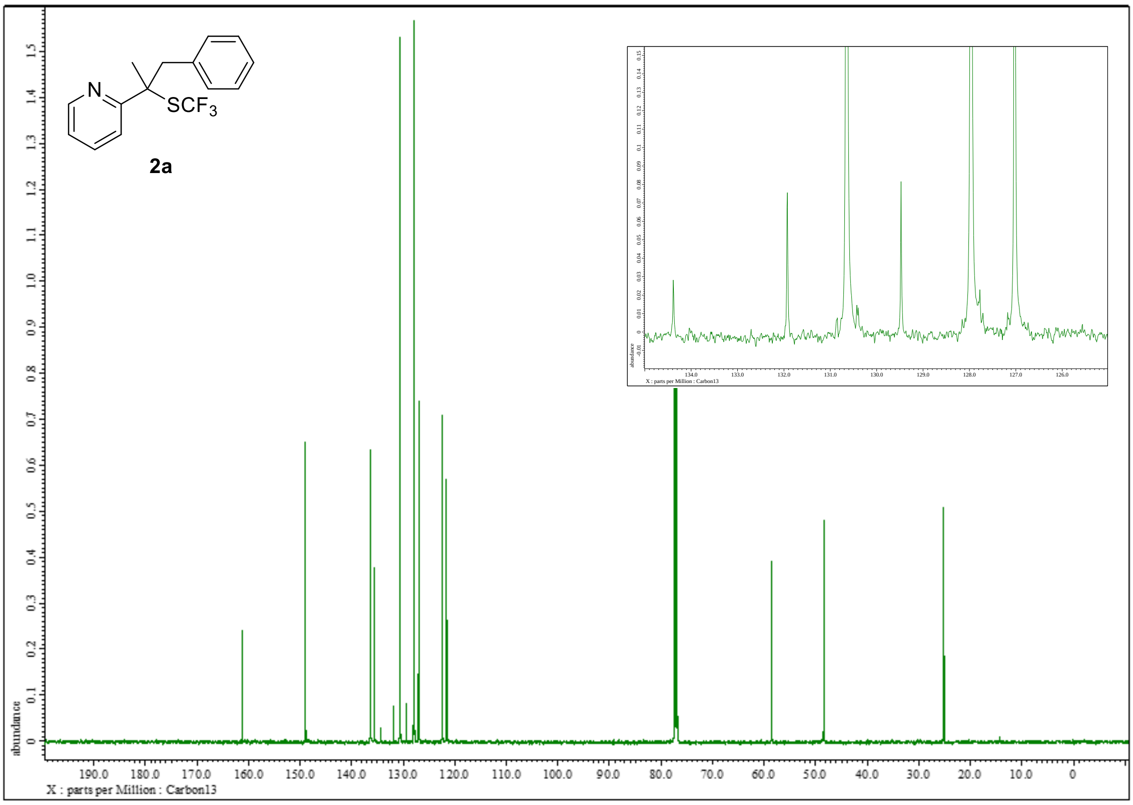
2a
X : parts per Million : Carbon13
abundance

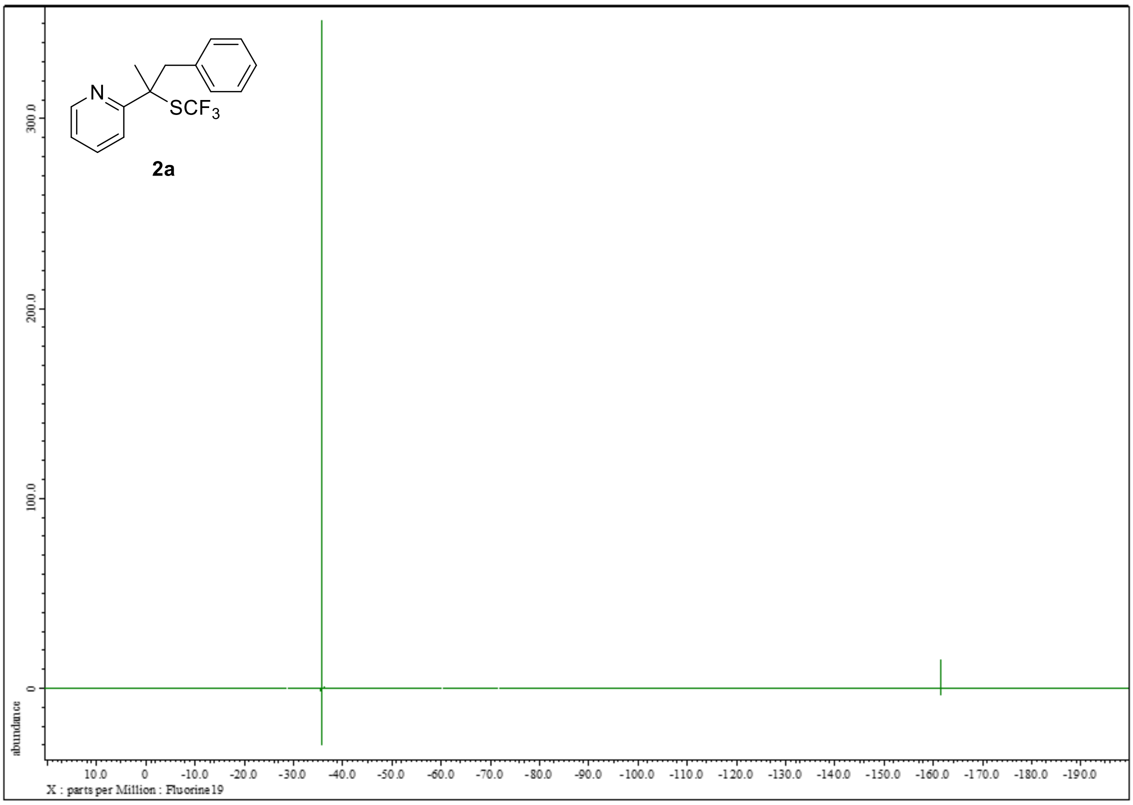
N
SCF3
2a
300.0
200.0
100.0
0
abundance
10.0
0
-10.0
-20.0
-30.0
-40.0
-50.0
-60.0
-70.0
-80.0
-90.0
-100.0
-110.0
-120.0
-130.0
-140.0
-150.0
-160.0
-170.0
-180.0
-190.0
X : parts per Million : Fluorine19

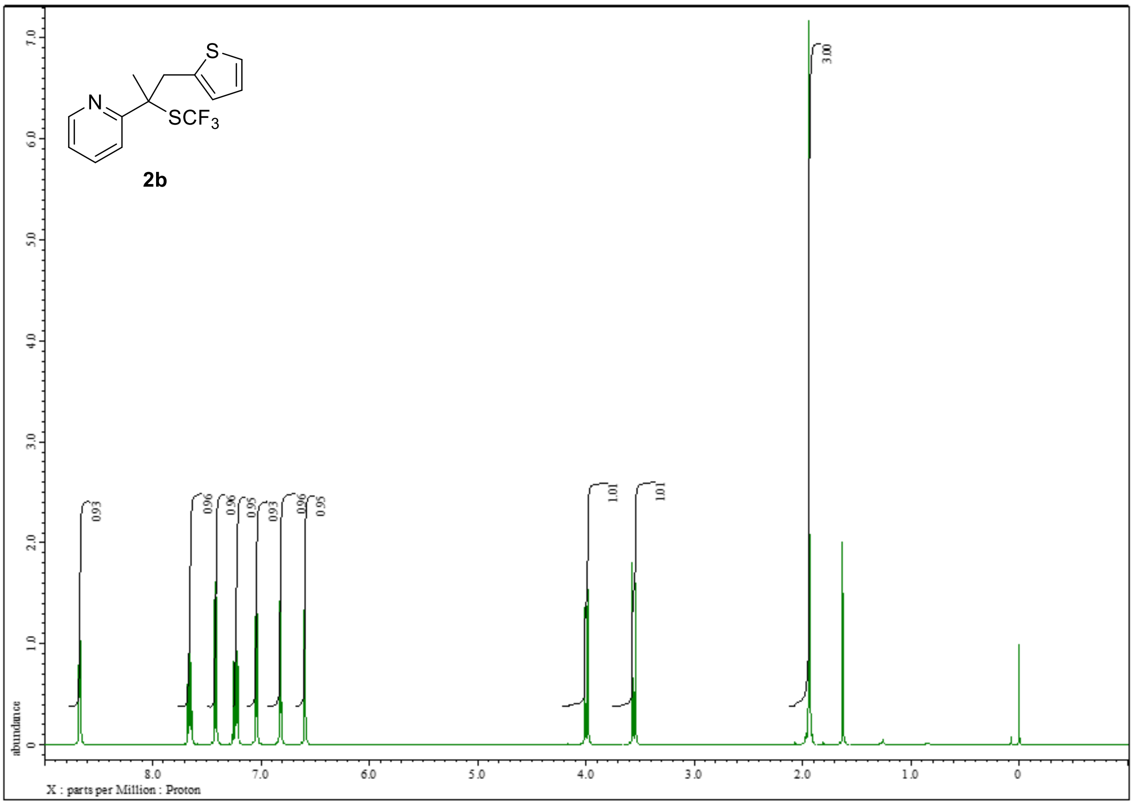

2b
SCF3
N
S
X : parts per Million : Proton
abundance
0.93
0.96
0.96
0.95
0.93
0.96
0.95
1.01
1.01
3.00


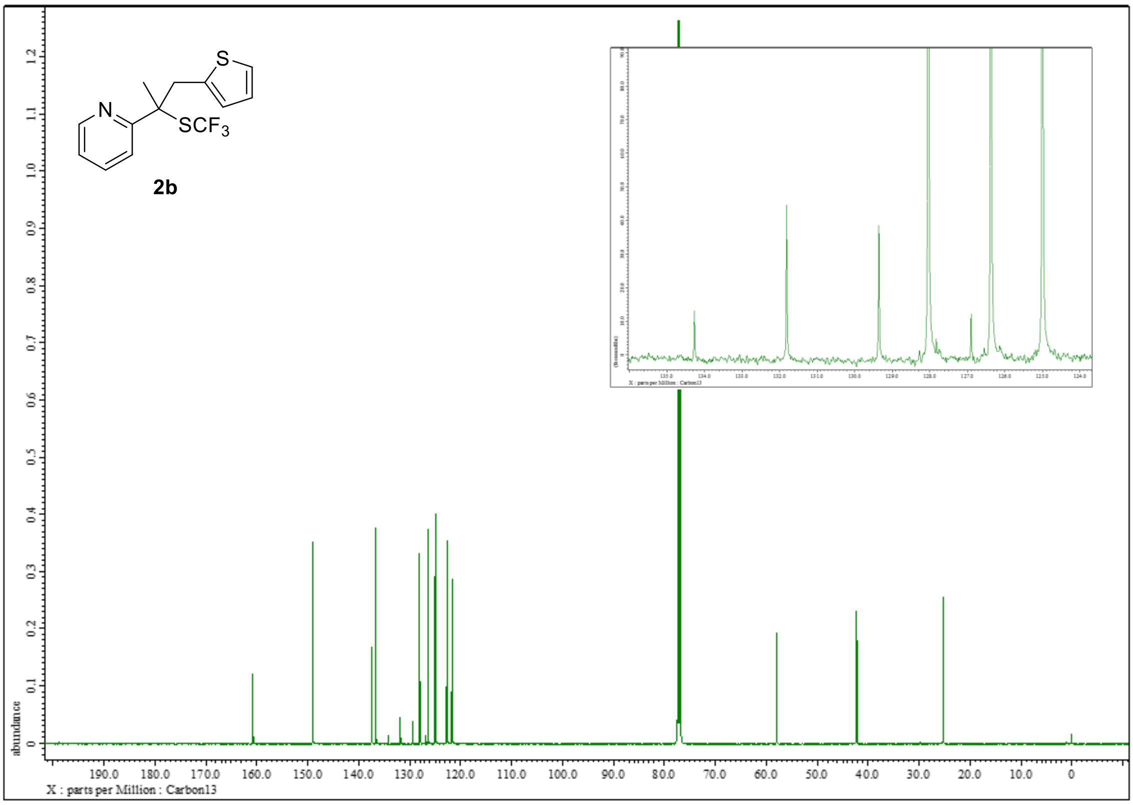

2b
SCF3
N
S
X : parts per Million : Carbon13
abundance

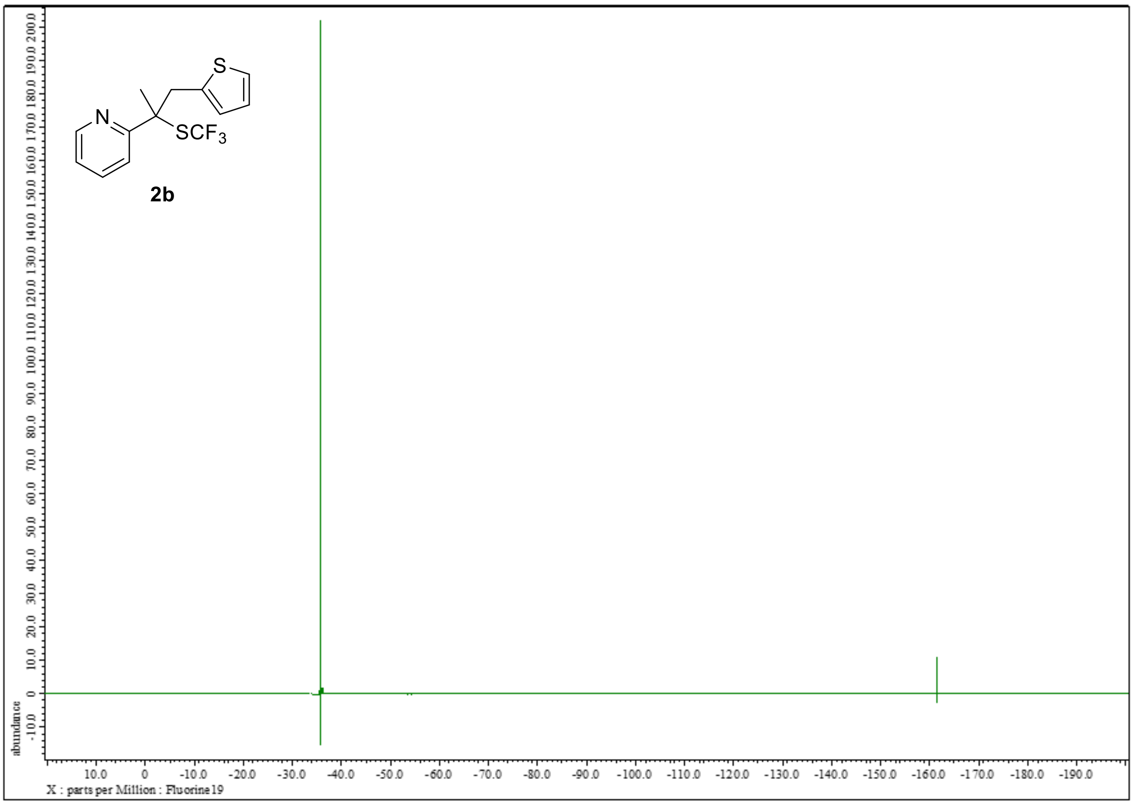

N
S
SCF3
2b
abundance
X : parts per Million : Fluorine19

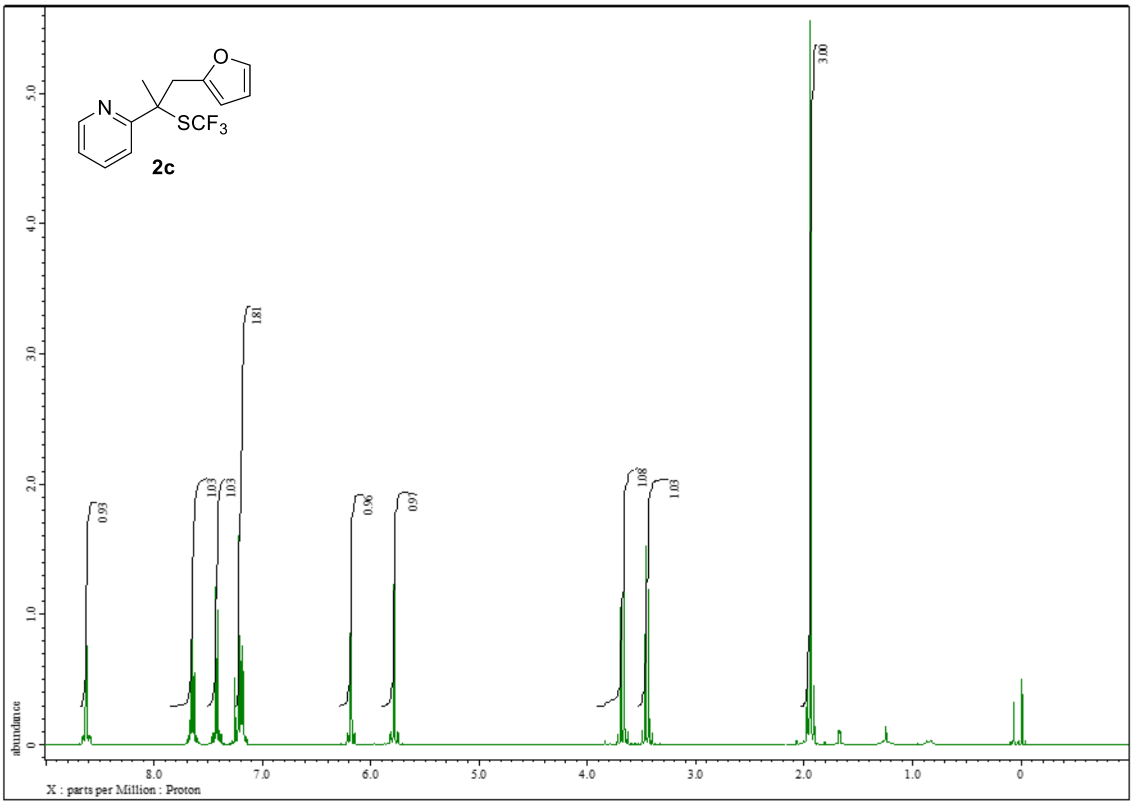

SCF3
2c
0.93
1.03
1.03
1.81
0.96
0.97
1.08
1.03
3.00
abundance
X : parts per Million : Proton


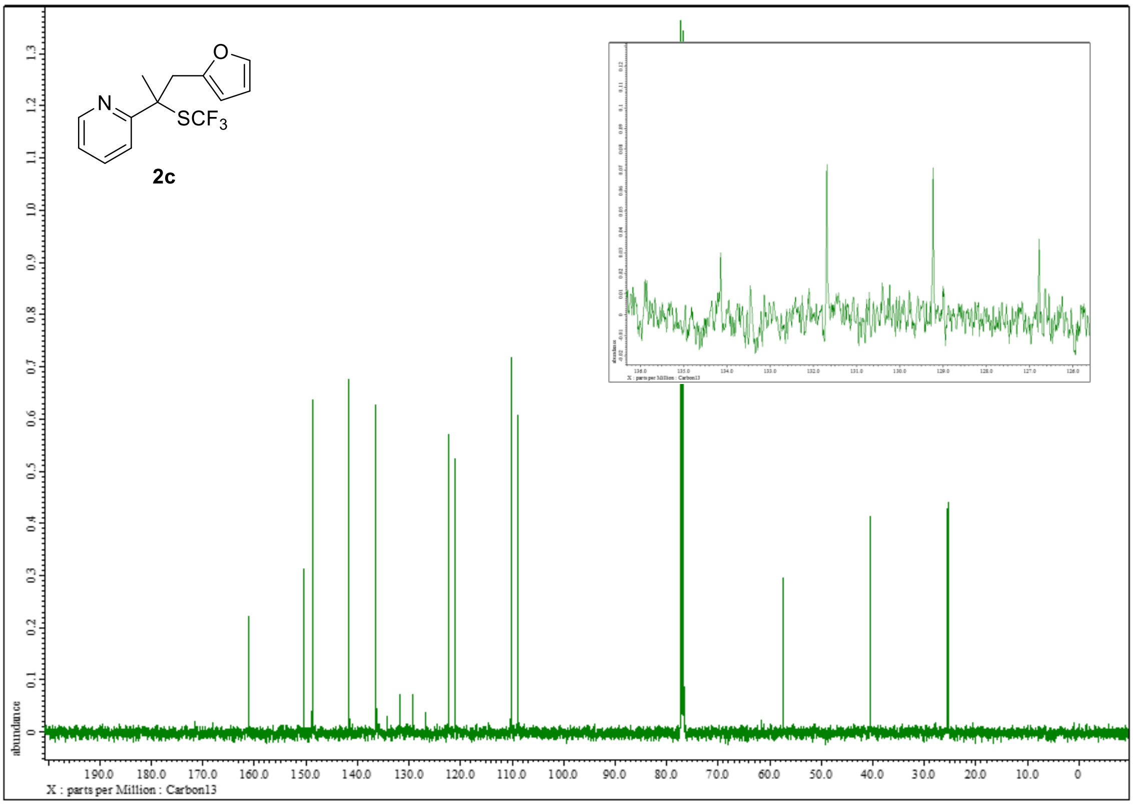

SCF3
2c
abundance
X : parts per Million : Carbon13

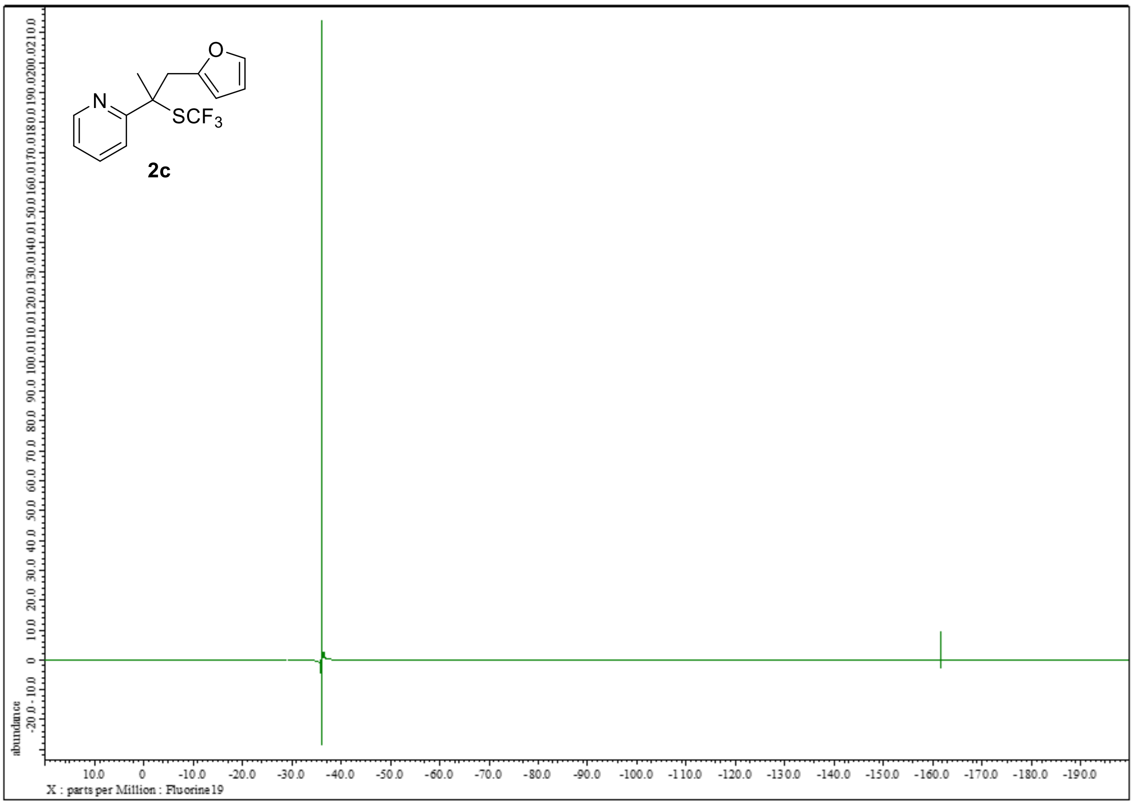
SCF3
2c
X : parts per Million : Fluorine19
abundance

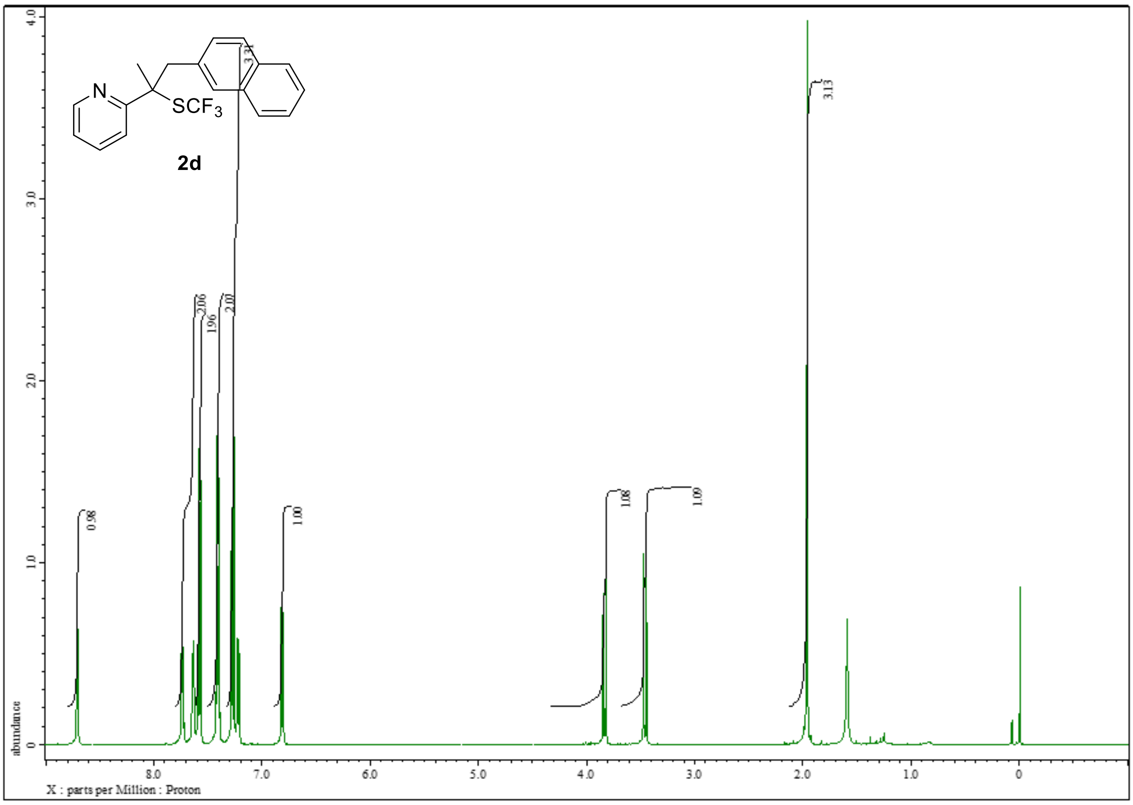
SCF3
2d
abundance
X : parts per Million : Proton

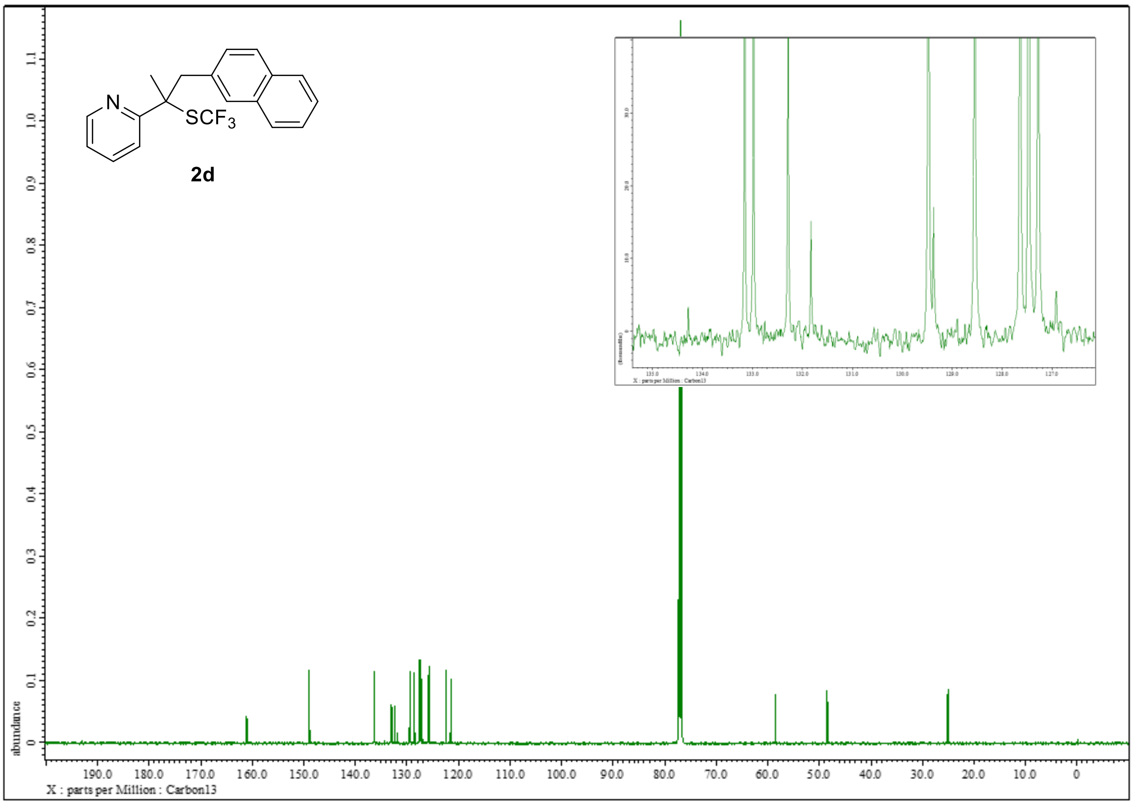
SCF3
2d
abundance
X : parts per Million : Carbon13

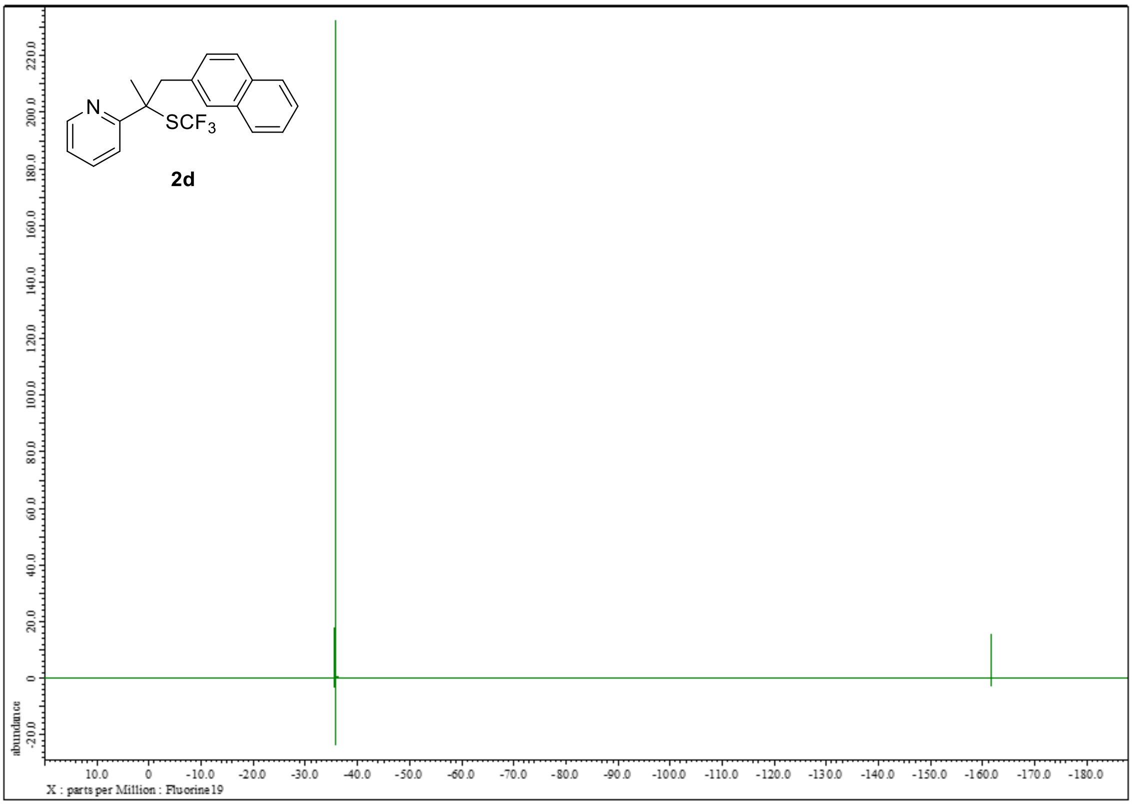
N
SCF$_3$
2d
abundance
X : parts per Million : Fluorine19

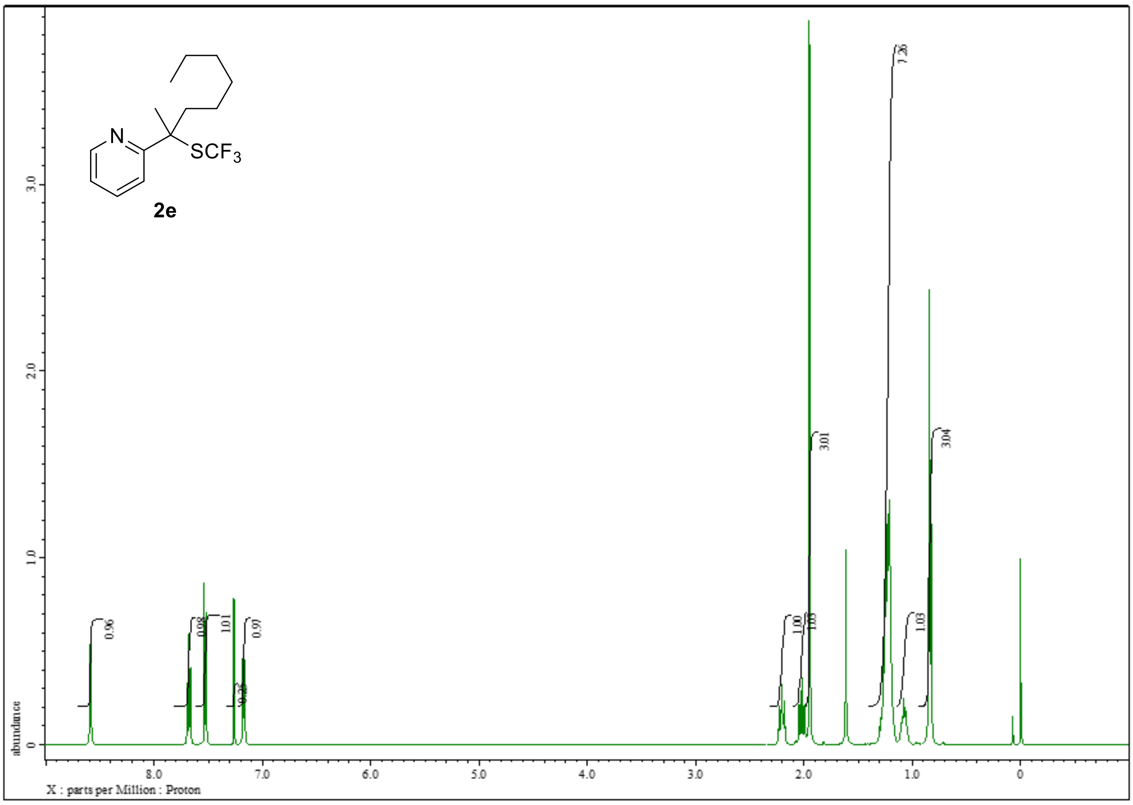
SCF3
2e
abundance
X : parts per Million : Proton

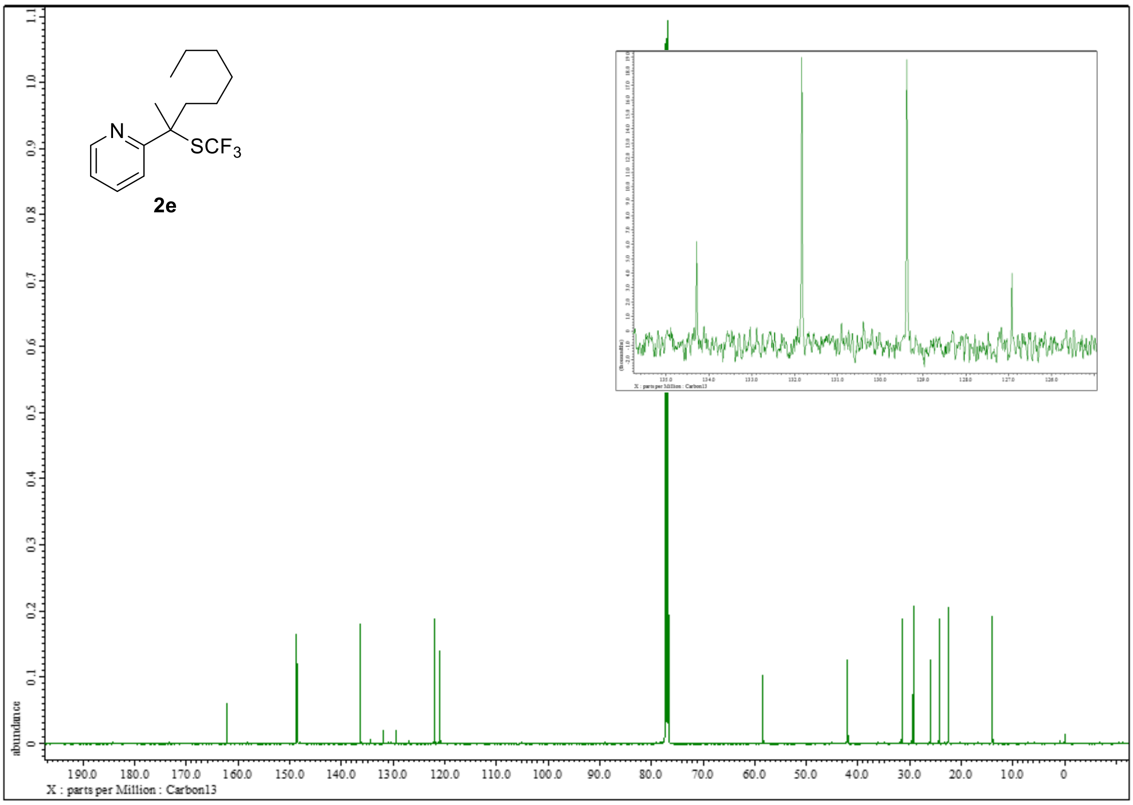
SCF3
2e
abundance
X : parts per Million : Carbon13

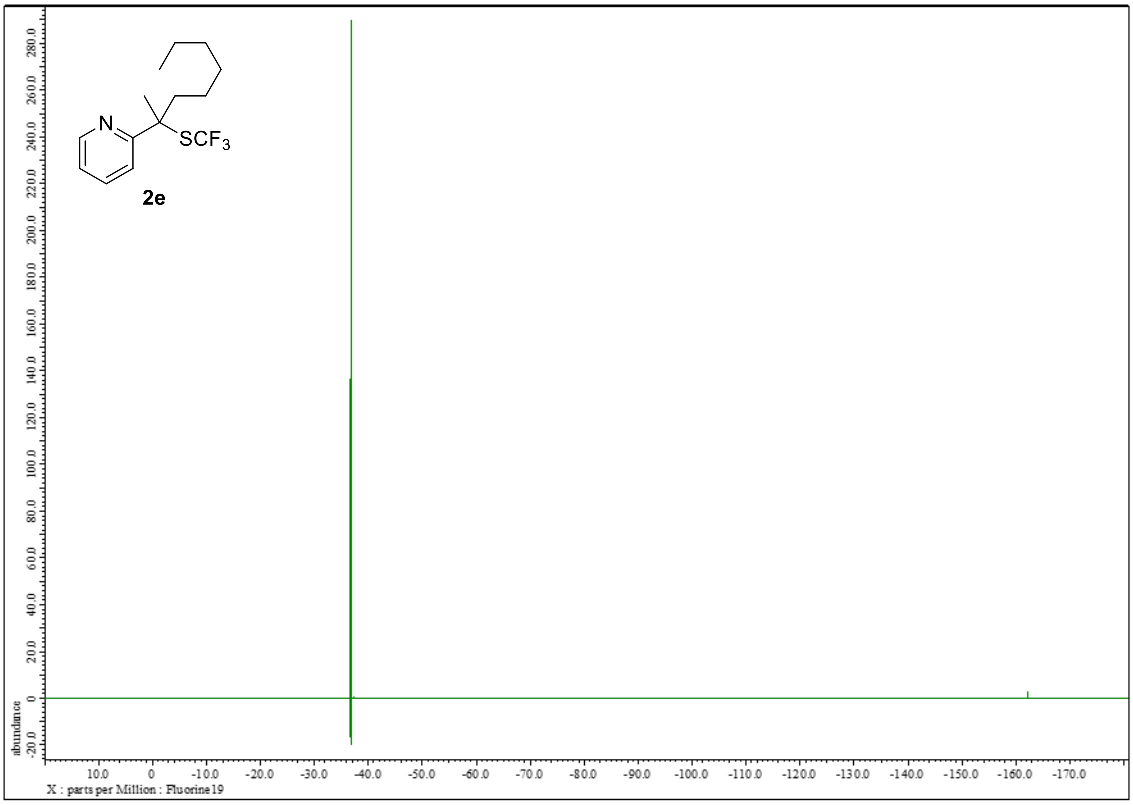
2e
SCF3
N
abundance
X : parts per Million : Fluorine19

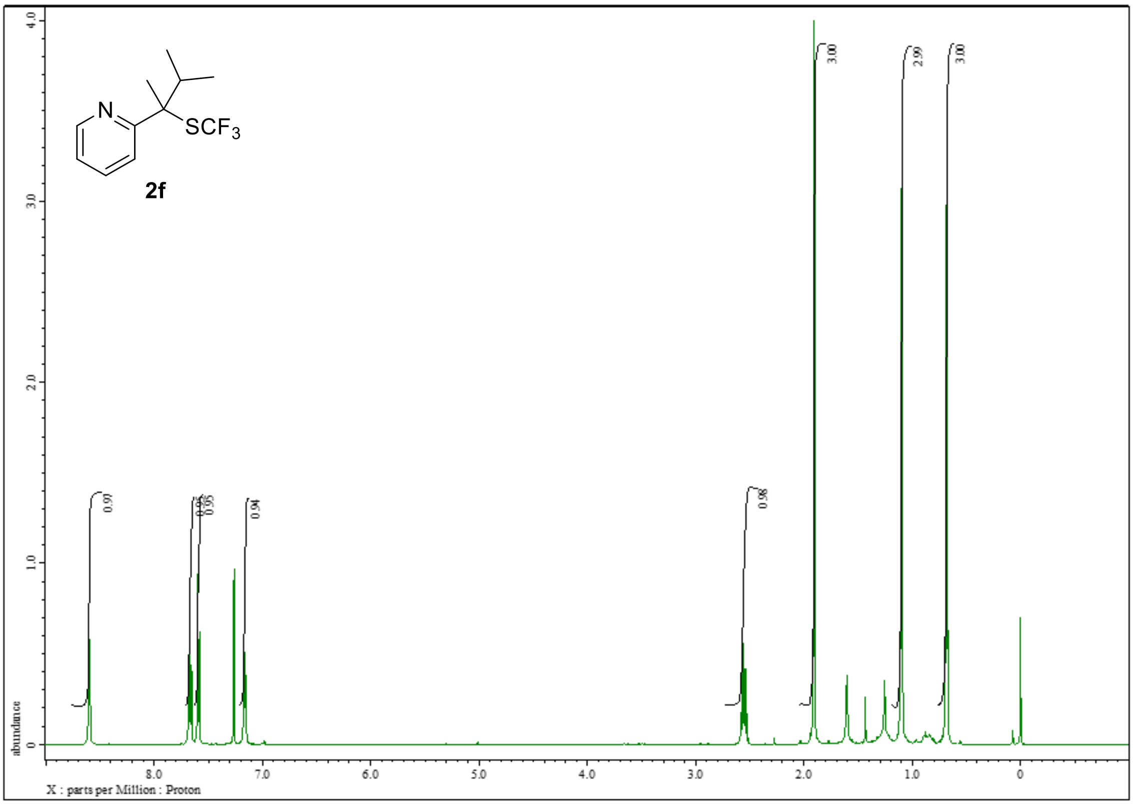

N
SCF3
2f
abundance
X : parts per Million : Proton
0.99
0.95
0.95
0.94
0.98
3.00
2.99
3.00


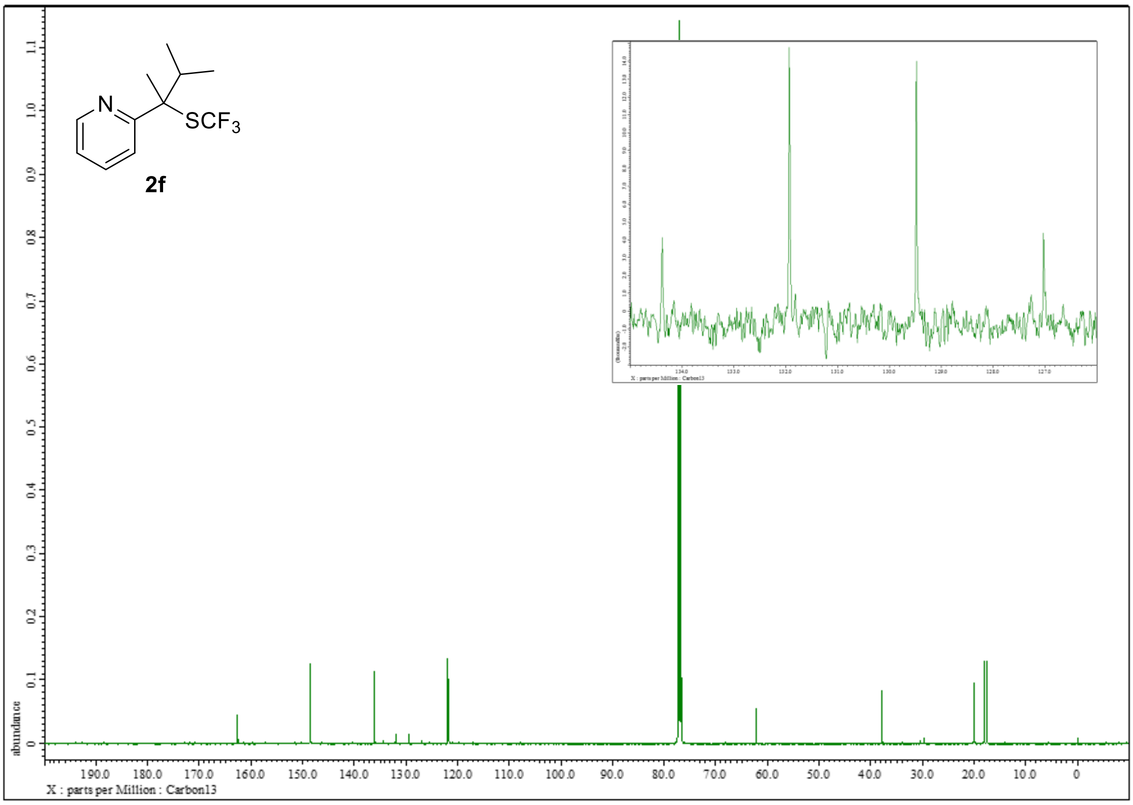

N
SCF3
2f
abundance
X : parts per Million : Carbon13

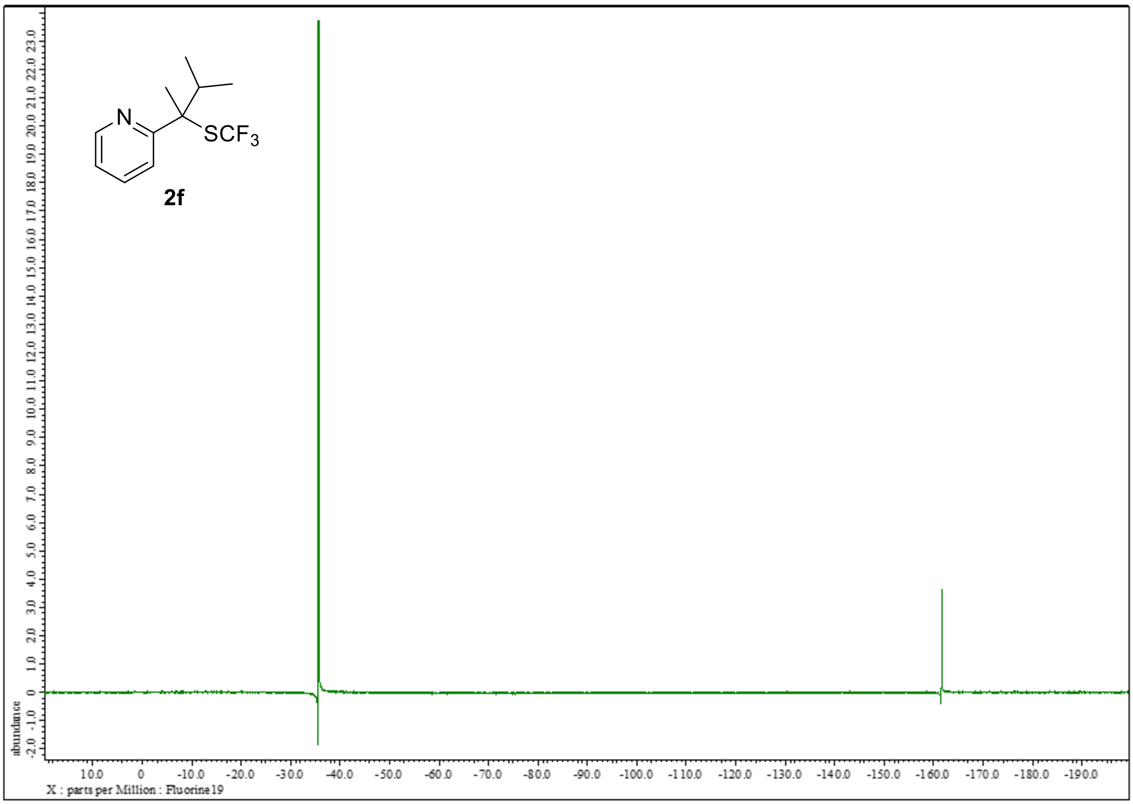

N
SCF3
2f
abundance
X : parts per Million : Fluorine19

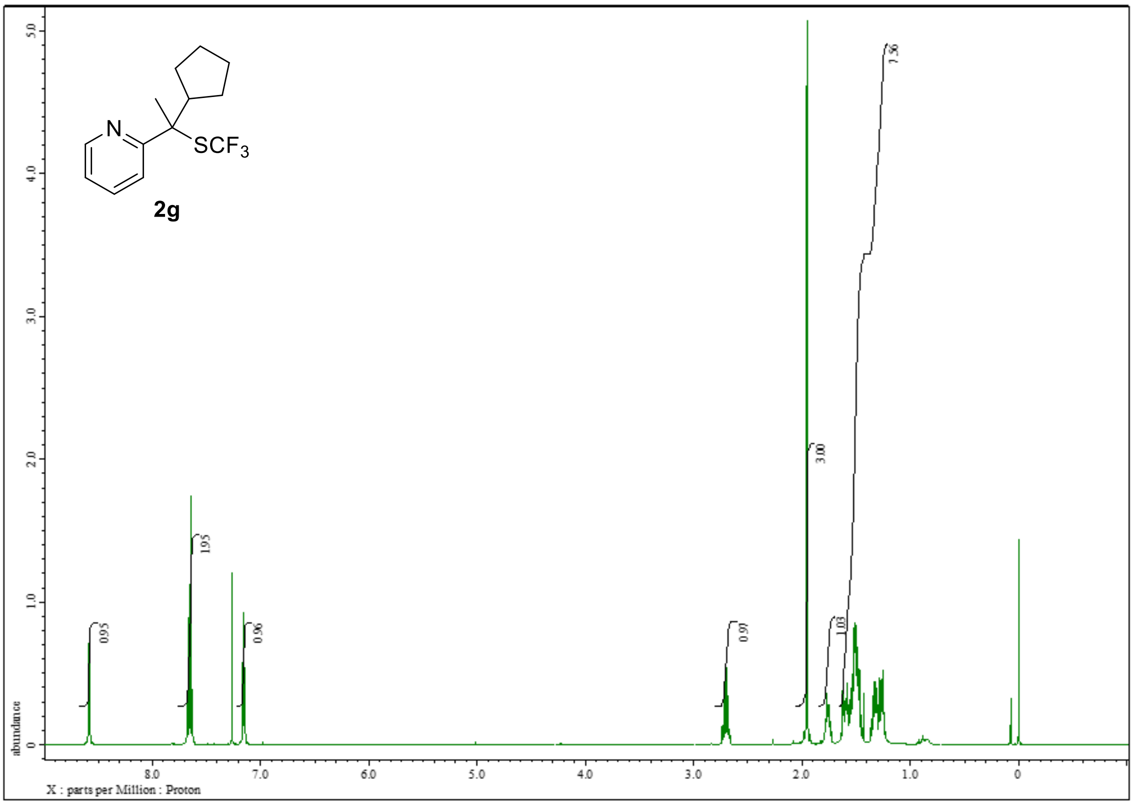

N
SCF3
2g
0.95
1.95
0.96
0.97
3.00
1.03
7.56
abundance
8.0
7.0
6.0
5.0
4.0
3.0
2.0
1.0
0
X : parts per Million : Proton


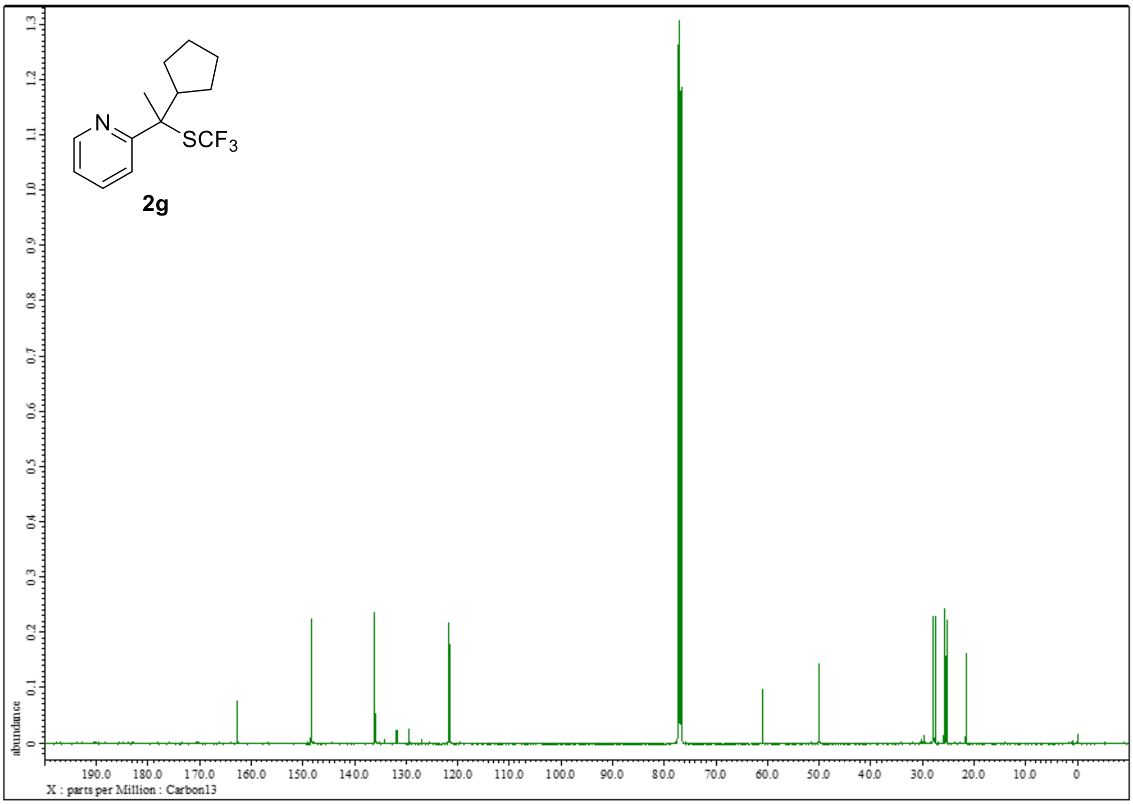

N
SCF3
2g
abundance
190.0
180.0
170.0
160.0
150.0
140.0
130.0
120.0
110.0
100.0
90.0
80.0
70.0
60.0
50.0
40.0
30.0
20.0
10.0
0
X : parts per Million : Carbon13

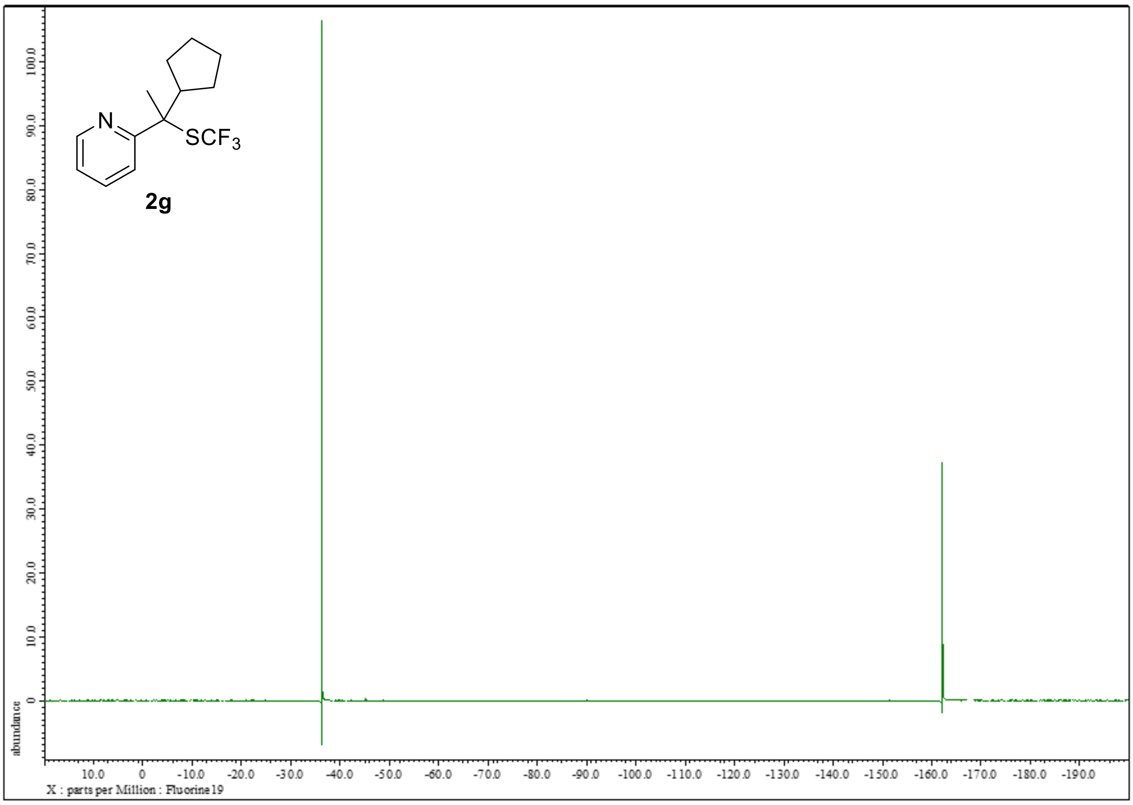

N
SCF3
2g
abundance
0
10.0
20.0
30.0
40.0
50.0
60.0
70.0
80.0
90.0
100.0
10.0
0
-10.0
-20.0
-30.0
-40.0
-50.0
-60.0
-70.0
-80.0
-90.0
-100.0
-110.0
-120.0
-130.0
-140.0
-150.0
-160.0
-170.0
-180.0
-190.0
X : parts per Million : Fluorine19

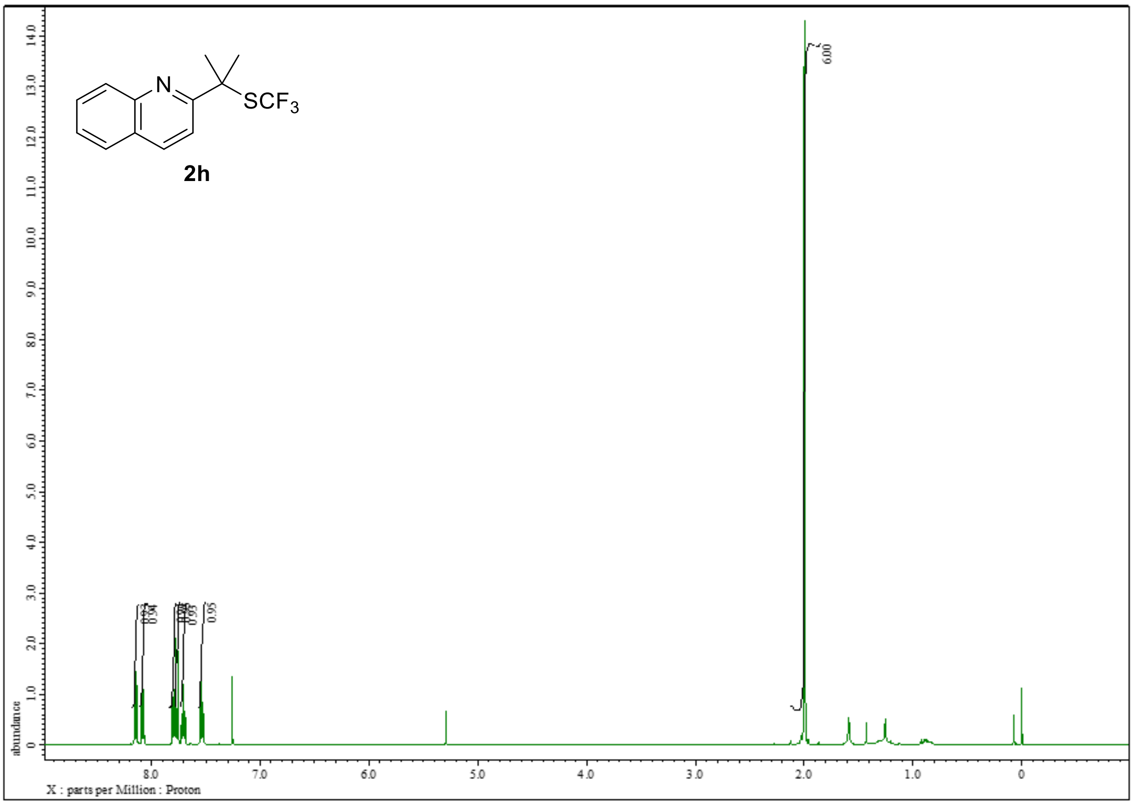
N
SCF3
2h
6.00
0.95
abundance
X : parts per Million : Proton

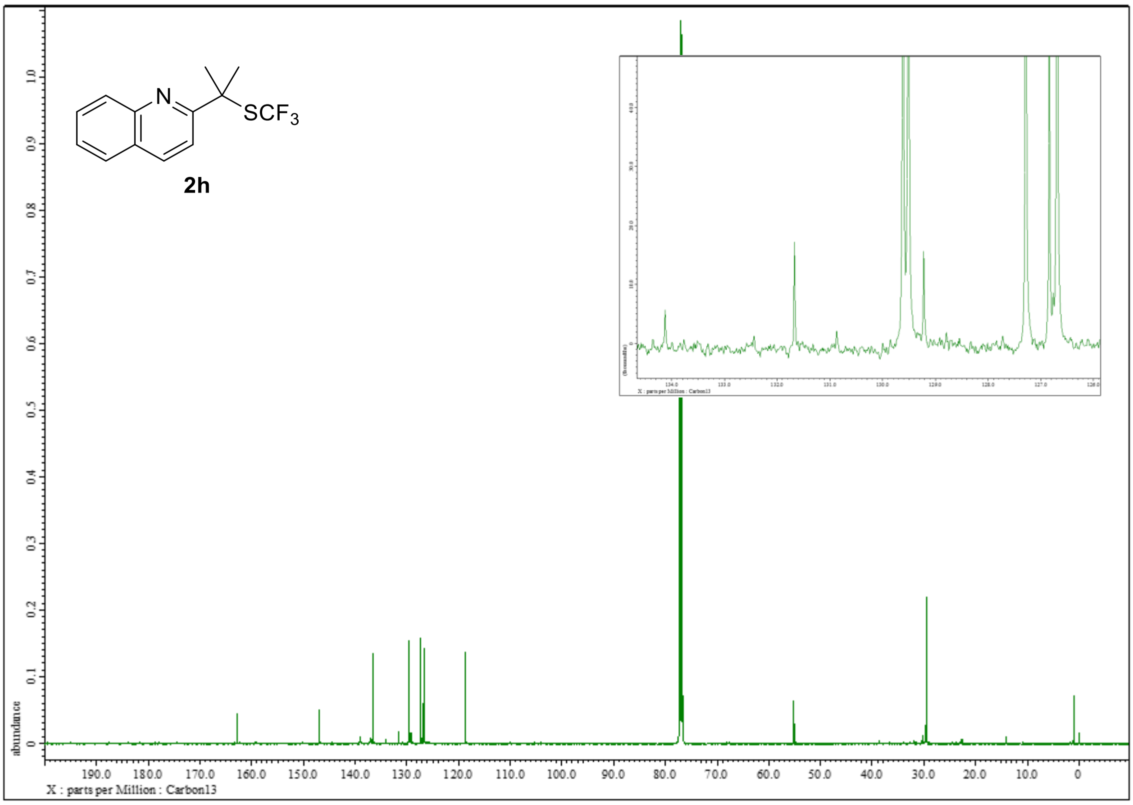
N
SCF3
2h
abundance
X : parts per Million : Carbon13

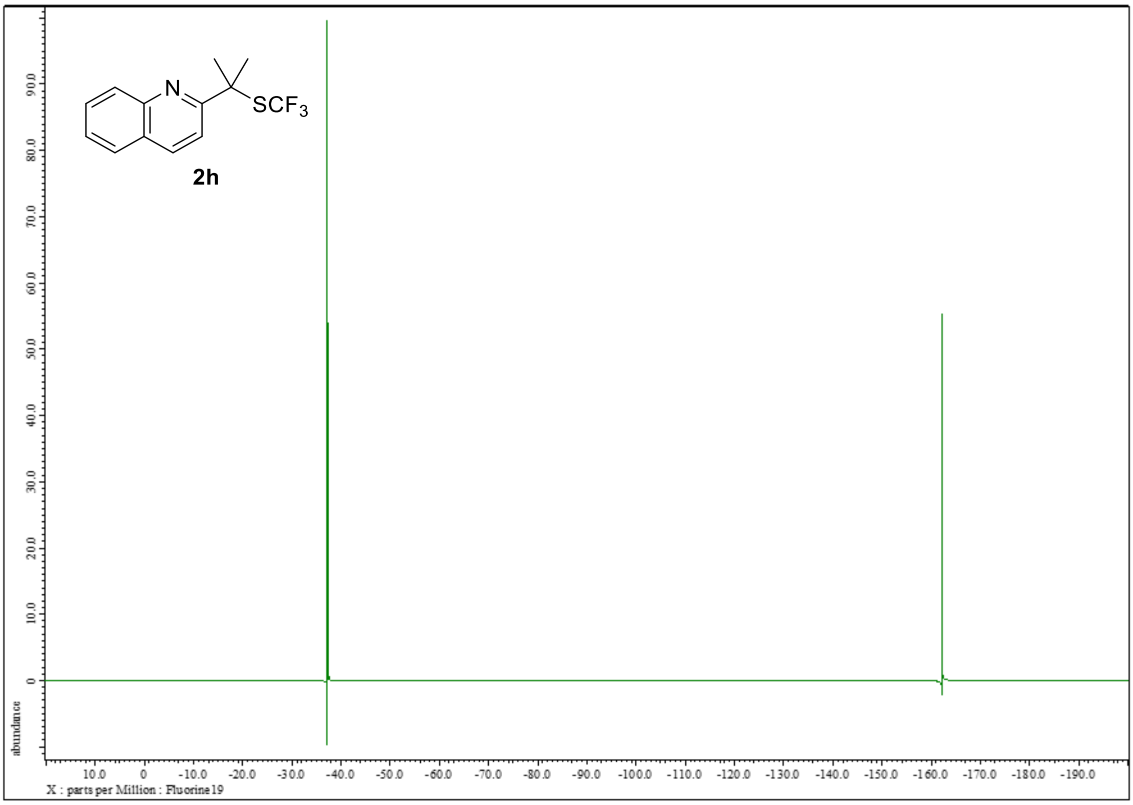

N
SCF3
2h
abundance
X : parts per Million : Fluorine19

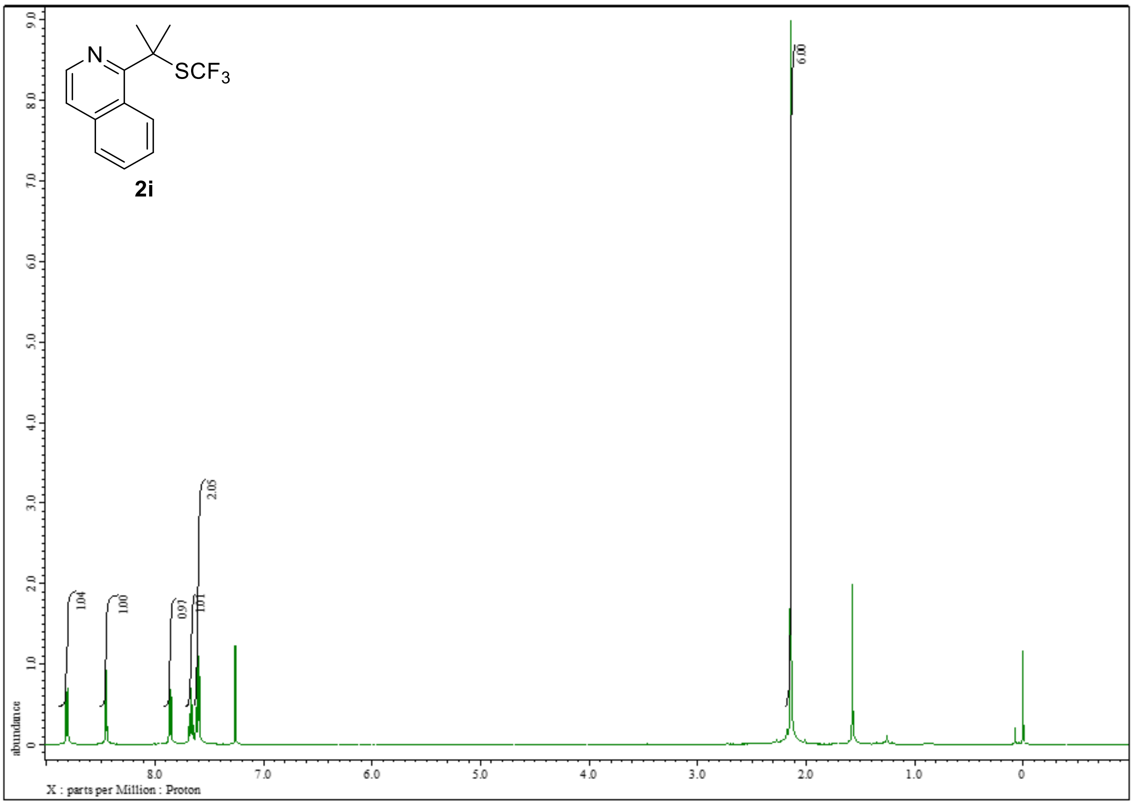
N
SCF3
2i
1.04
1.00
0.97
1.01
2.05
6.00
abundance
X : parts per Million : Proton

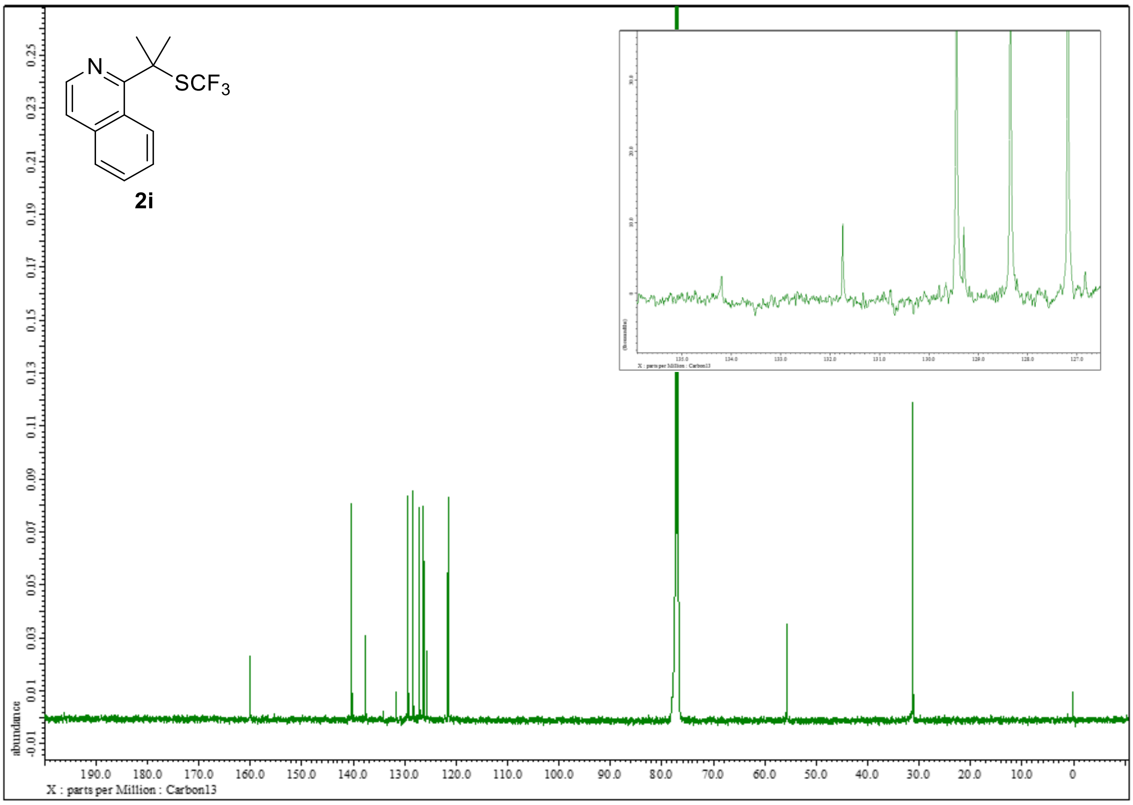
N
SCF3
2i
abundance
X : parts per Million : Carbon13

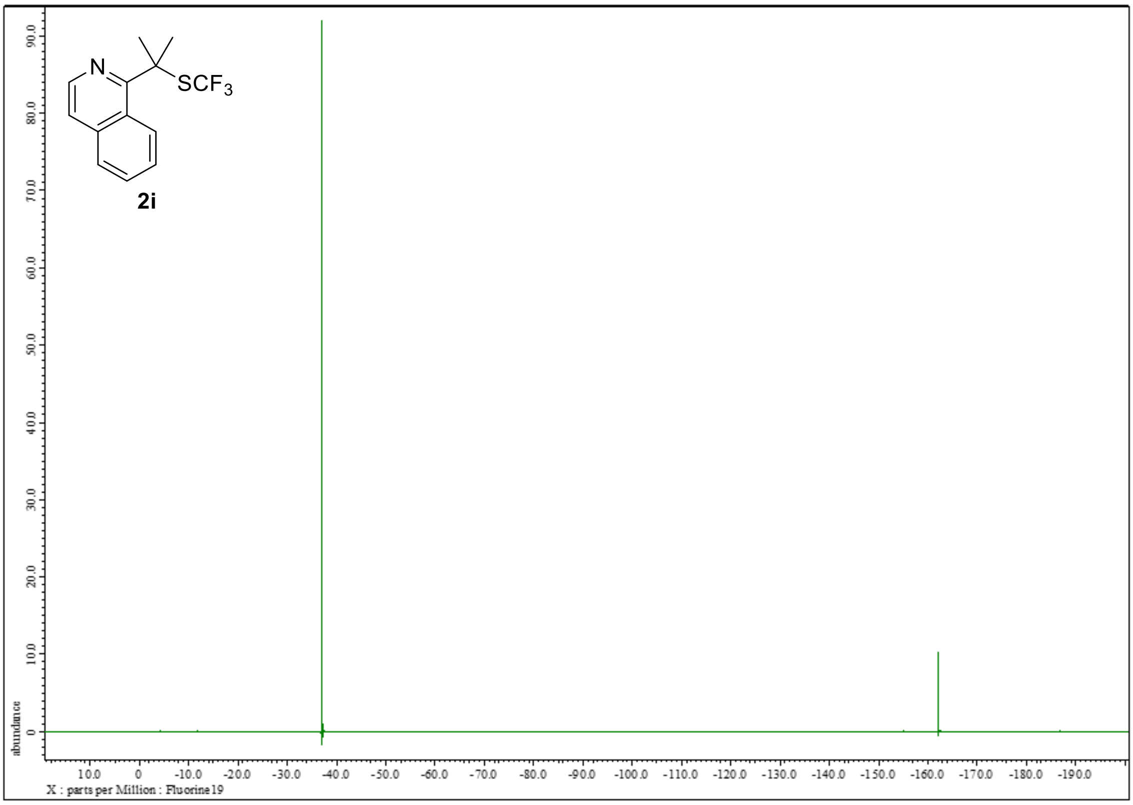

N
SCF3
2i
abundance
X : parts per Million : Fluorine19

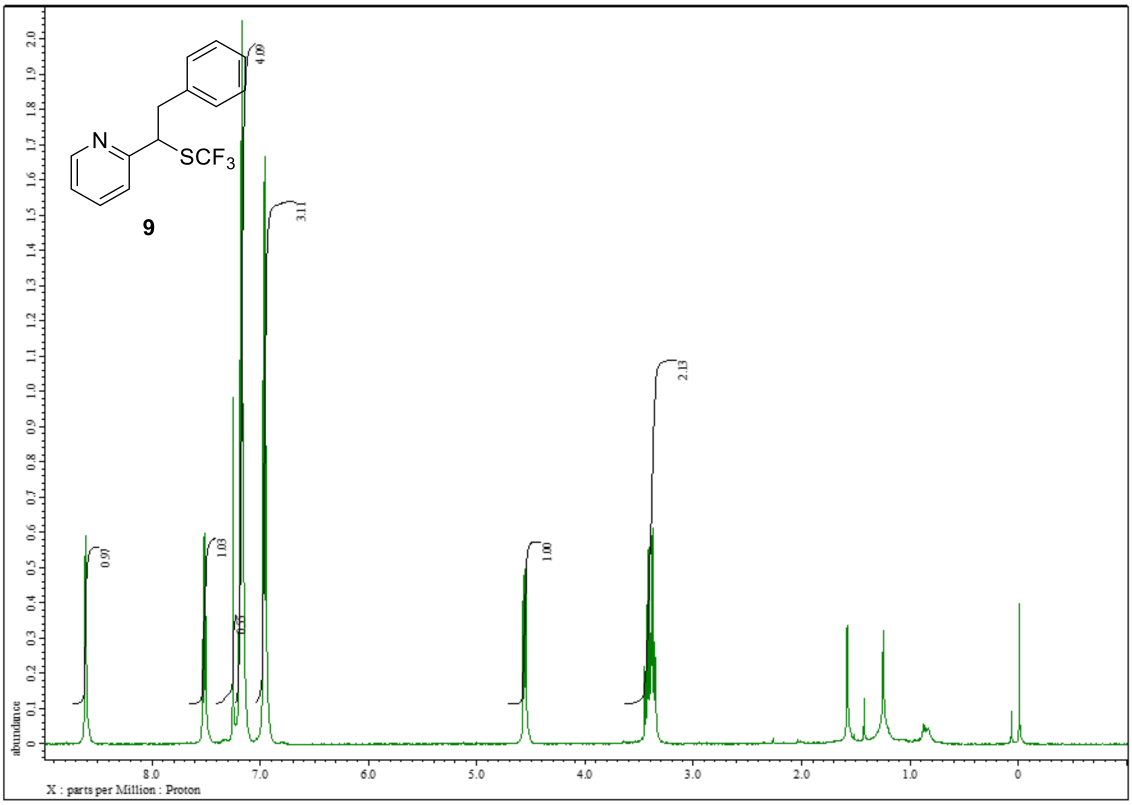
N
SCF3
9
abundance
0.97
1.03
4.09
3.11
1.00
2.13
8.0
7.0
6.0
5.0
4.0
3.0
2.0
1.0
0
X : parts per Million : Proton

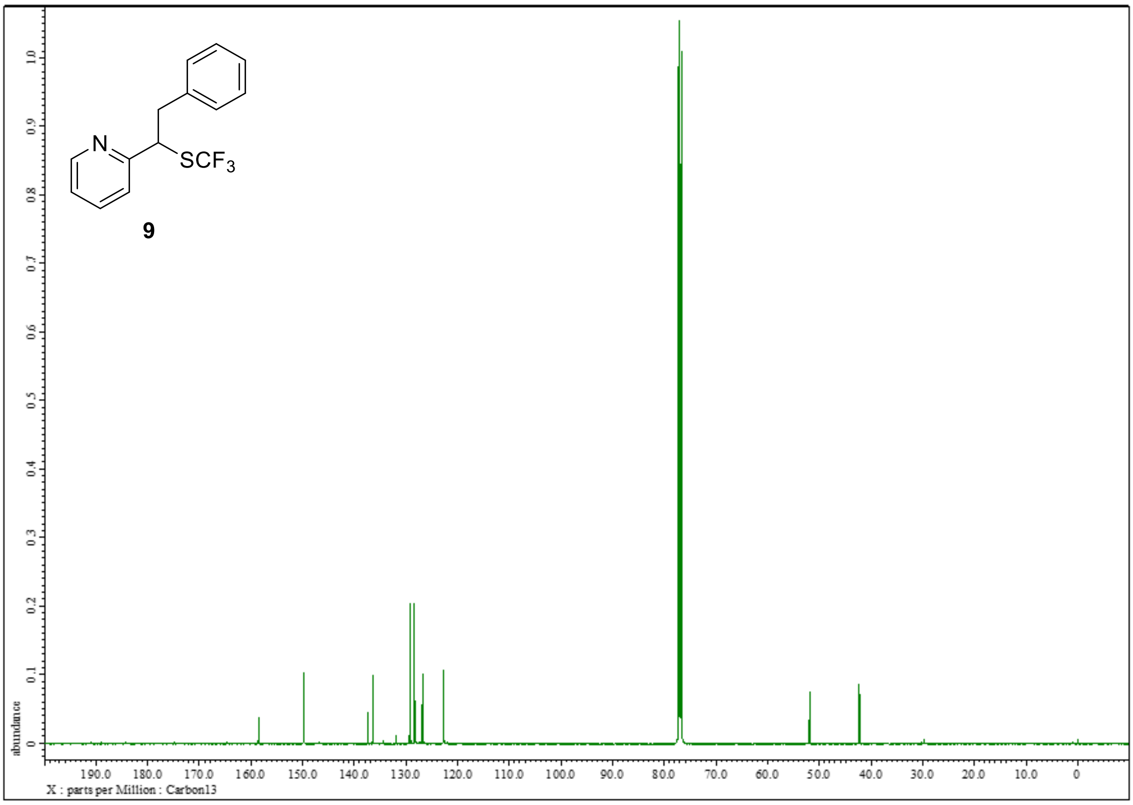
N
SCF3
9
abundance
190.0
180.0
170.0
160.0
150.0
140.0
130.0
120.0
110.0
100.0
90.0
80.0
70.0
60.0
50.0
40.0
30.0
20.0
10.0
0
X : parts per Million : Carbon13

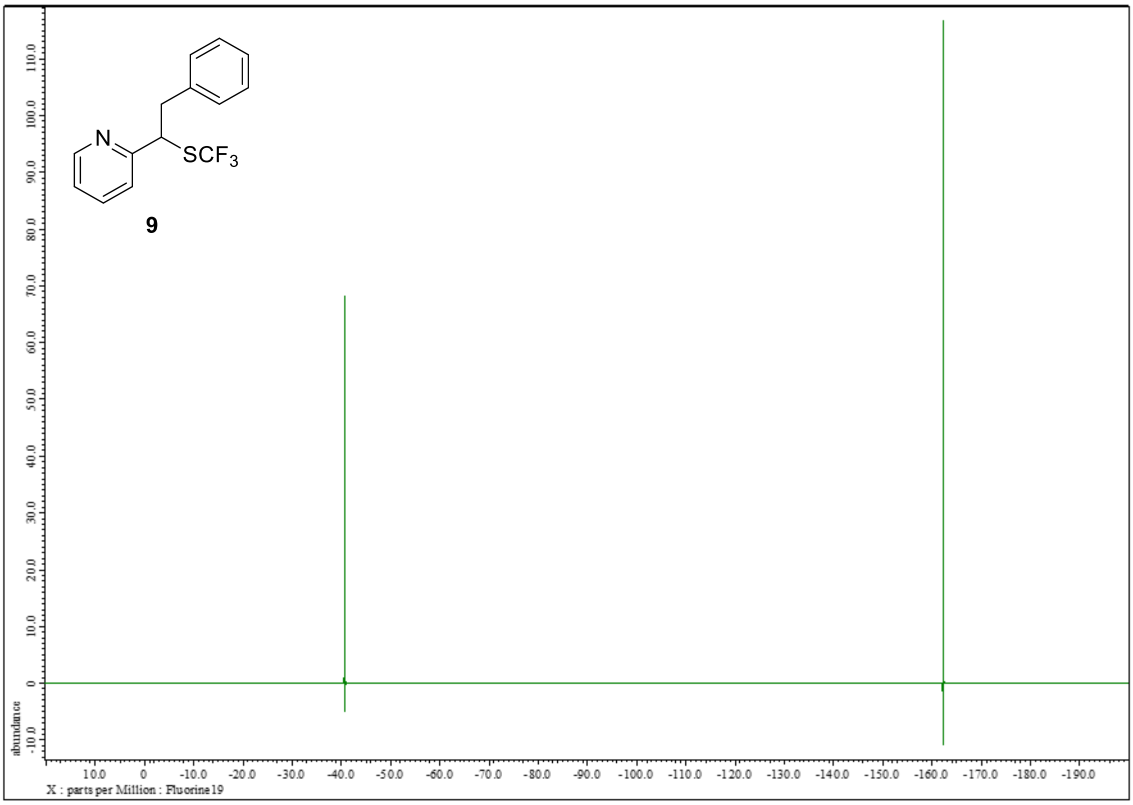

SCF3
9
abundance
X : parts per Million : Fluorine19

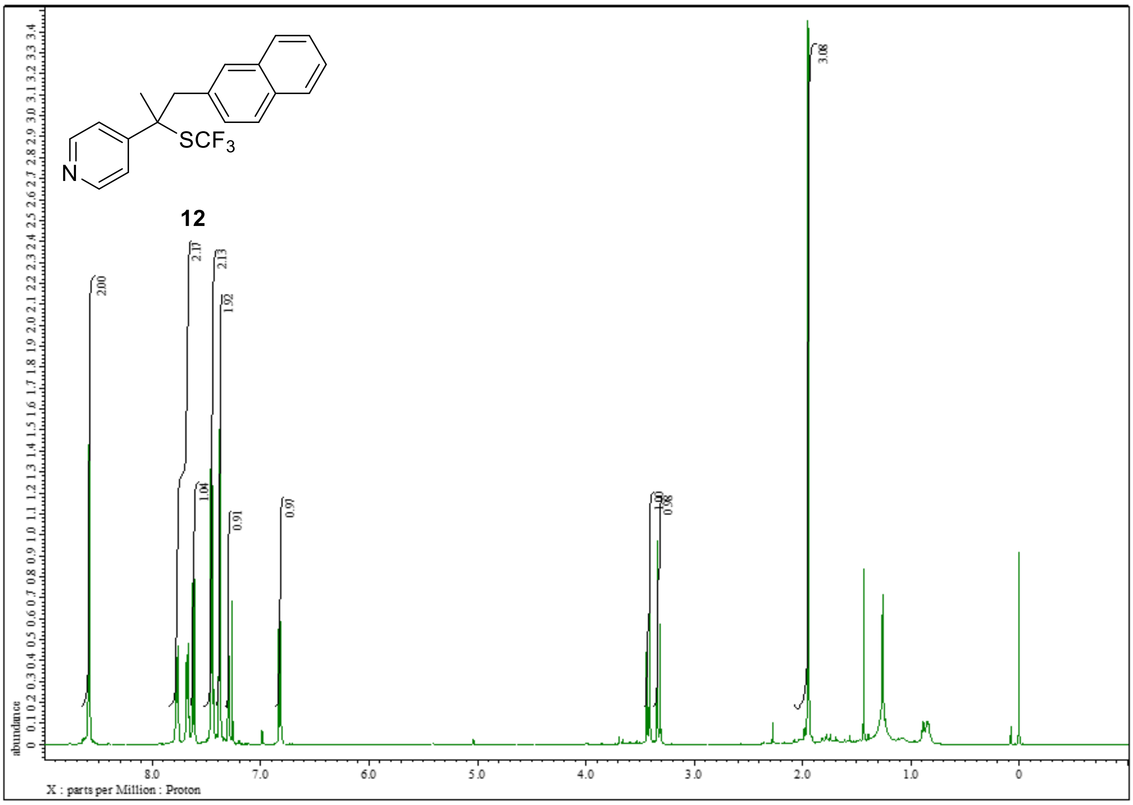
SCF3
N
12
X : parts per Million : Proton
abundance

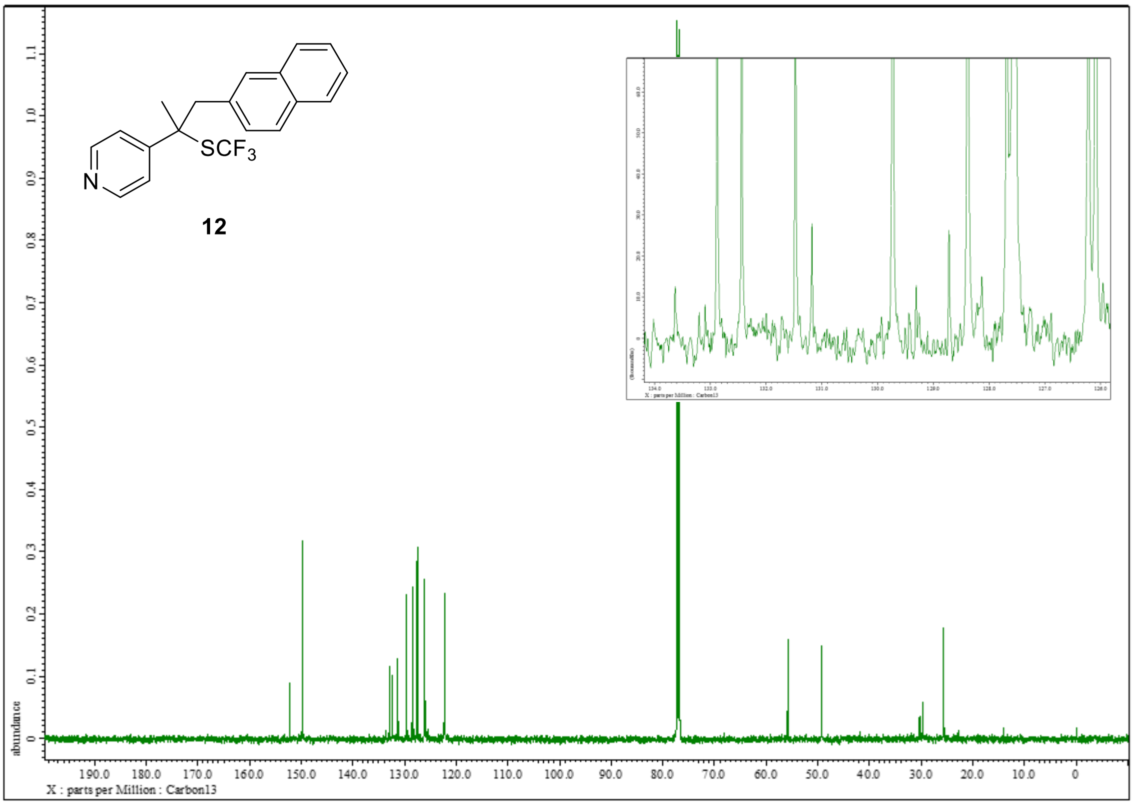
SCF3
N
12
X : parts per Million : Carbon13
abundance

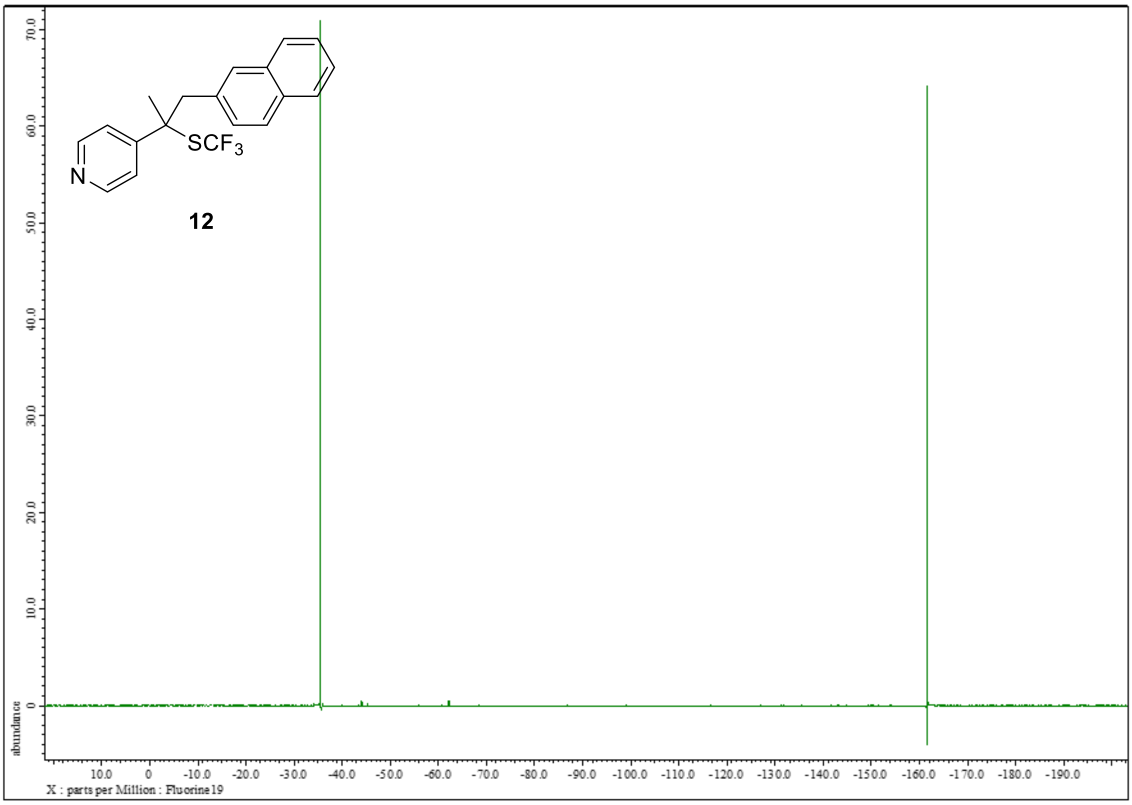
SCF3
N
12
70.0
60.0
50.0
40.0
30.0
20.0
10.0
0
abundance
10.0
0
-10.0
-20.0
-30.0
-40.0
-50.0
-60.0
-70.0
-80.0
-90.0
-100.0
-110.0
-120.0
-130.0
-140.0
-150.0
-160.0
-170.0
-180.0
-190.0
X : parts per Million : Fluorine19
